# PiT2 regulates neuronal outgrowth through interaction with microtubule-associated protein 1B

**DOI:** 10.1038/s41598-017-17953-3

**Published:** 2017-12-19

**Authors:** Xi-Xiang Ma, Xiangyang Li, Ping Yi, Cheng Wang, Jun Weng, Li Zhang, Xuan Xu, Hao Sun, Shenglei Feng, Kai Liu, Rui Chen, Shiyue Du, Xiao Mao, Xiaomei Zeng, Luo-Ying Zhang, Mugen Liu, Bei-Sha Tang, Xiaojuan Zhu, Shan Jin, Jing-Yu Liu

**Affiliations:** 1 Key Laboratory of Molecular Biophysics of the Ministry of Education, Center for Human Genome Research, College of Life Science and Technology, Huazhong University of Science and Technology (HUST), Wuhan, 430074 China; 2 College of Life Sciences, Hubei Collaborative Innovation Center for Green Transformation of Bio-Resources, Hubei University, Wuhan, 430062 China; 3 Department of Neurology, Xiangya Hospital, Central South University, Changsha, Hunan 410008 China; 4 College of Life Sciences, Northeast Normal University, Changchun, 130024 China

## Abstract

PiT2 is a member of the inorganic phosphate transporter family, and is extensively expressed in the nervous system. It was found that loop7 domain of PiT2 is not required for retroviral recognition and transport function. The exact functions of loop7 remain poorly understood. Here we show that loop7 of PiT2 is necessary for the transport of PiT2 protein to the cell surface. Further, loop7 is also related to the outgrowth of neurite in Neuro2A cells interacts with the light chain 1 of microtubule-associated protein 1B (MAP1B). PiT2 with mutated MAP1B binding sites affect neurite outgrowth whereas Pi transport function deficient mutants of PiT2 do not. We also show that *Drosophila* dPiT interacts with microtubule-associated protein Futsch, and dPiT is crucial for the normal development of neuromuscular junctions (NMJs). These results indicate that PiT2 might participate in the regulation of neuronal outgrowth by interacting with MAP1B and independently of its Pi transport function in the nervous system.

## Introduction

PiT2 is a member of the inorganic phosphate (Pi) transporter family (Transport Classification Database Number 2.A.20), and is encoded by *SLC20A2* ^1,2^. *SLC20A2* was cloned by Van Zeijl *et al*. in 1994 and was found to be comprised of 11 exons^3^. PiT2 possesses dual functions including as retroviral receptor GLVR2 or Ram-1 (Murine leukemia virus receptor)^4–6^ and sodium-dependent phosphate transporter^7,8^. As a member of the Na^+^-coupled mammalian type-III inorganic phosphate (Pi) transporters, PiT2 transports inorganic phosphate across the cell membrane against a chemical and electrical gradient^2,9^. In the mouse brain, PiT2 is mainly expressed in neurons, and is also known to be expressed in astrocytes and vascular endothelial cells^10–12^. Our recent study linked *SLC20A2* to familial idiopathic basal ganglia calcification (IBGC). Loss-of-function mutations in *SLC20A2* result in calcium phosphate deposition due to regional Pi accumulation in extracellular matrix of the brain^13^.

PiT2 consists of 652 amino acids^14^. According to bioinformatics predictions, cysteine scanning, epitope tagging and *in vitro* glycosylation studies, the topological model of PiT2 has 12 transmembrane domains (TMDs) with extracellular N- and C-terminal tails, 2 ProDom domains I_11_-L_161_ (N-PD1131) and V_492_-V_640_ (C-PD1131) located in the N-terminal and C-terminal of PiT2 respectively^14,15^. Corresponding to the protein functions of PiT2, loop regions in PD domain, such as 67–91, 107–141, 517–530 amino acid residues are required for amphotropic murine leukemia virus (A-MuLV) binding^16,17^, and PD domains also play an important role in maintaining transport function^18^. In IBGC families, 23 missense variants have been found in *SLC20A2*, and these missense variants are mainly located in two PD domains of PiT2^19^. The PiT2 also contains a 246-aa (about 38 percent amino acids of PiT2) large intracellular loop7 domain between N-PD1131 and C-PD1131^20^. Bøttger and Pedersen had reported that the PiT2 with deleted loop7 had normal retroviral recognition, and transport functions^15^. So far, there is no definite evidence that missense variants in loop7 affect the transport function of PiT2 which result in IBGC^19^. Therefore, it remains an intriguing question regarding the function of loop7 domain in the nervous system.

To investigate possible functions of loop7 domain of PiT2 in the nervous system, we conducted immunofluorescence assays of Neuro2A cells transfected with PiT2 that lack loop7, and found that loop7 deletion affected the subcellular localization of PiT2 protein and neurite outgrowth in Neuro2A cells. To reveal the function of loop7 in PiT2, we performed yeast two-hybrid screening and identified microtubule-associated protein 1B (MAP1B) as a novel interactor of loop7 in PiT2. MAP1B is first synthesized and then cleaved to generate a heavy chain (HC) and a light chain (LC)^21^. As a cytoskeletal protein that regulates actin and microtubule dynamics, MAP1B plays important roles in axonal elongation and regeneration, neuronal migration, axonal guidance, dendritic spine morphology, as well as expression, trafficking and activity of neurotransmitter receptors^22,23^. Differentiation assay showed that MAP1B binding site mutants of PiT2 decreased the length of neurites in Neuro2A cells. In *Drosophila*, *CG42575* (encoding dPiT protein) is homologous to human *SLC20A2*, and there is only one representative of MAP1 family: the *futsch* gene^24^. Futsch protein is cleaved similarly to MAP1 proteins in vertebrates^25^. Futsch is also implicated in neuronal development^26,27^. To dissect the neuronal function of loop7 domain *in vivo*, we generated transgenic lines that could be used to tissue-specifically overexpress dPiT with or without loop7. We performed co-immunoprecipitation and confirmed the interaction between *Drosophila* dPiT and Futsch. Immunochemical analyses showed that dPiT was crucial for the normal development of neuromuscular junctions (NMJs). This study reveals a novel function of PiT2 in neuronal outgrowth by interacting with MAP1B *in vivo* and *in vitro*.

## Results

### The loop7 domain is essential for PiT2 localization and might impact neurite outgrowth in Neuro2A cells

**Figure 1:**
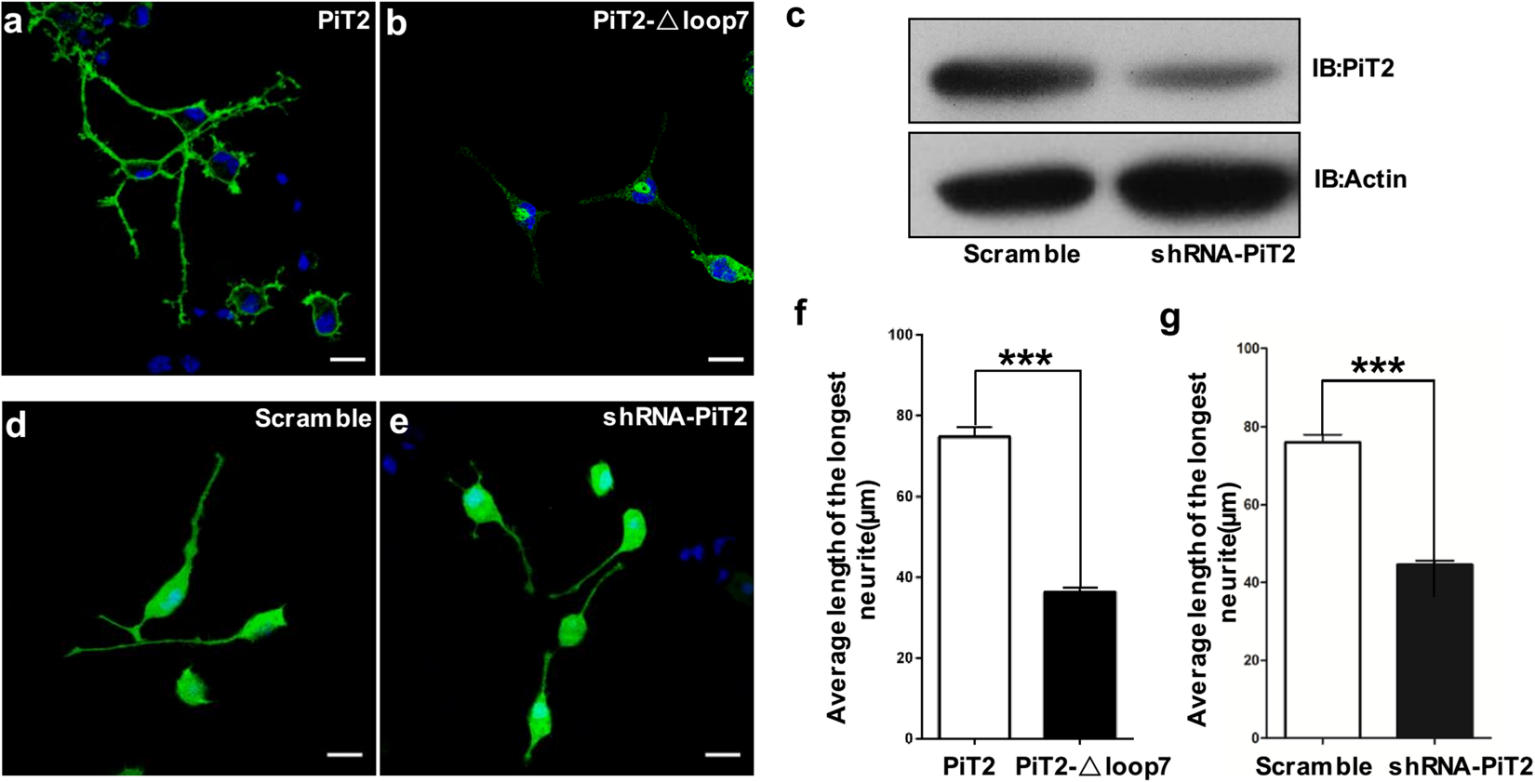
The loop7 domain of PiT2 impacts neurite outgrowth in Neuro2A cells. (**a**,**b**) Differentiated Neuro2A cells stained with anti-HA/Alexa Flour488 (green) and DAPI (blue) with overexpression of HA-tagged wild type PiT2 (**a**) or HA-tagged PiT2-Δloop7 (**b**). Scale bar, 20 μm. (**c**) Neuro2A cells were transiently transfected with pSIH-PiT2 or pSIH-scramble. Cell lysates were immunoblotted with anti-PiT2 and anti-actin antibodies. Full length blots are shown in Supplementary Fig. S2. (**d**,**e**) Differentiated Neuro2A cells stained with DAPI (blue) with transfection of pSIH-scramble (**d**) or pSIH-PiT2 (**e**). Scale bar, 20 μm. (**f**) Quantification of neurite length of differentiated Neuro2A cells transfected with wild type PiT2 or PiT2-Δloop7 plasmids. (**g**) Average length of the longest neurite of Neuro2A cells transfected with scramble and shRNA-PiT2 were statistically analyzed. Error bars show the mean ± s.e.m. of 100 randomly selected cells from each group in three independent experiments. *** means P < 0.001.

To get precise information about loop7 function in the nervous system, we first performed immunofluorescence assays of Neuro2A cells transfected with wild-type (PiT2-WT) or loop7 deletion mutant, in which residues 254–483 of PiT2 were deleted (PiT2-Δloop7). The PiT2-WT proteins were localized on plasma membranes in undifferentiated (Supplementary Fig. S1a) and differentiated Neuro2A cells (Fig. 1a), but most of the PiT2-Δloop7 proteins were found in the cytoplasm, and aggregated in a specific region of the cytoplasm (Fig. 1b, Supplementary Fig. S1c). These findings indicated that loop7 might be necessary for trafficking of PiT2 protein to the cell surface. In differentiated Neuro2A cells transfected with PiT2-Δloop7, we observed that deletion of loop7 induced a decrease in neurite length compared with Neuro2A cells transfected with WT (Fig. 1a,b,f). To further explore the biological function of loop7 in Neuro2A cell differentiation, we performed neuritogenesis assay. Following induction of differentiation by retinoic acid (RA) treatment, lengthening of Neuro2A cell neurites were detected. Knockdown of PiT2 by shRNA-PiT2 significantly decreased the length of the longest neurites by about one half compared with negative control (Fig. 1c–e,g and Supplementary Fig. S2). These results indicate that PiT2 might participate in the growth and development of the nervous system.

### Identification of MAP1B as a novel interaction partner of PiT2 by yeast two-hybrid screening

**Figure 2:**
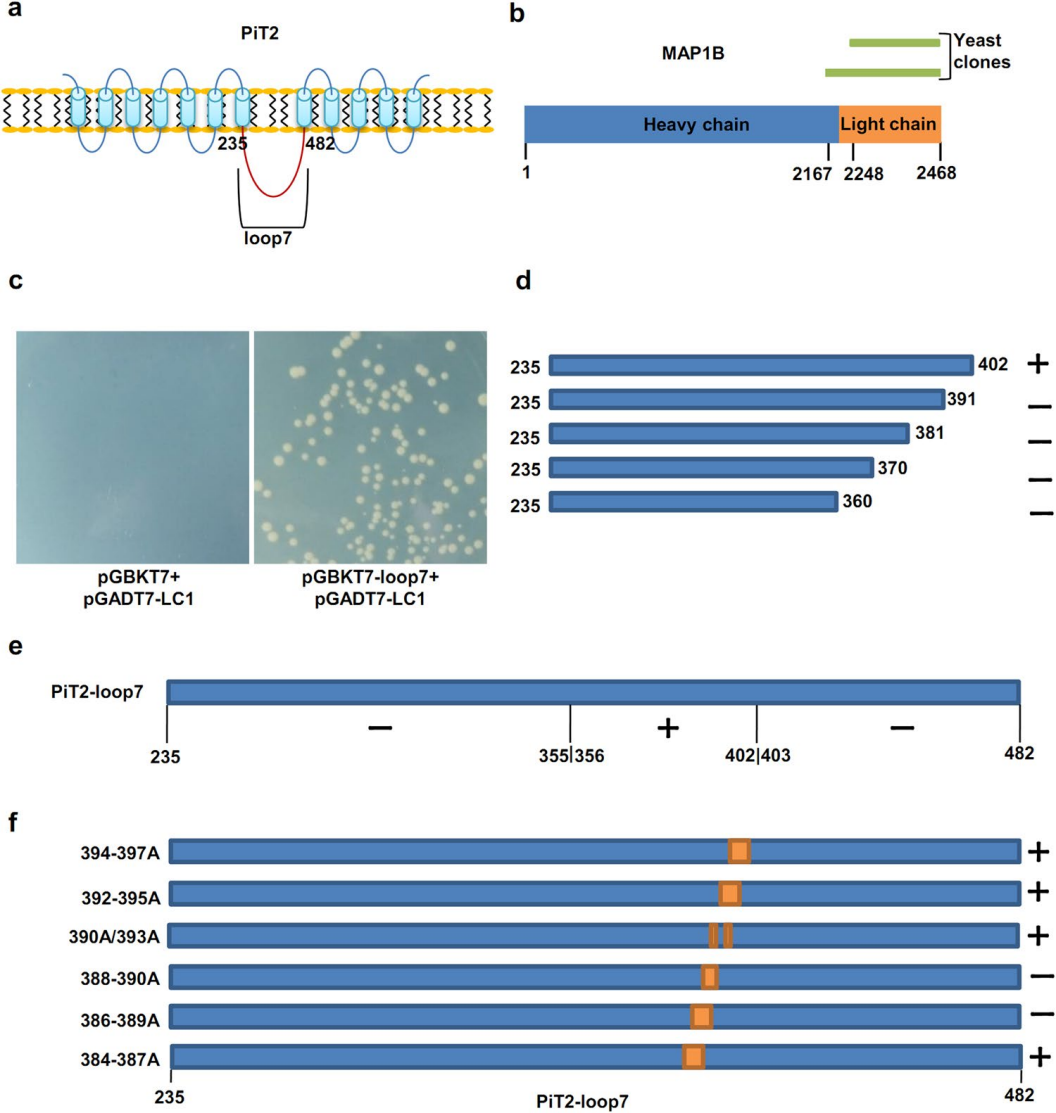
Yeast two-hybrid screen for the interacting protein of PiT2, and localization of MAP1B interaction site within loop7 of PiT2. (**a**) Schematic representation of PiT2, loop7 domain (residues 235–482, marked in red) was used as the bait for the yeast two-hybrid screen. (**b**) Schematic of the two yeast clones of MAP1B identified in the yeast two-hybrid screen. (**c**) Reconfirmation of the interaction between MAP1B and PiT2 in yeast. The transformants co-transformed with light chain of MAP1B and loop7 domain of PiT2 showed significant growth on SD/–Ade/–His/–Leu/–Trp selection agar plates compared with negative control. (**d**) Five C-terminal deletion mutants of loop7 were assayed for interaction with MAP1B using the yeast two-hybrid, only residues 235–402 interacted with LC1. (**e**) Loop7 truncation mutants were examined for interaction with MAP1B by the yeast two-hybrid assay, and only yeasts co-transformed with residues 356–402 and LC1 constructs showed growth on selection agar plates. (**f**) Alanine-scanning constructs designed to narrow the domains involved in interaction between residues 384 and 397. Mutations of residues 386–389 and 388–390 prevented the interaction between PiT2 and MAP1B. +, growth on stringent selection plates; −, no growth.

To search for interaction proteins involved in the subcellular localization or neurite outgrowth regulation of PiT2, yeast two-hybrid screening was performed. Residues 235–482 (loop7 domain) were used as bait (Fig. 2a), and was fused to the Gal4 DNA-binding domain. Through mating of the fetal brain cDNA library and Y187/pGBKT7-loop7 we succeeded in screening approximately 400,000 independent clones. After selection of fetal brain cDNA library, 183 positive yeast clones showing His-reporter and Ade-reporter gene activity were selected. Further high-stringency selection and sequencing of the AD plasmid inserts led to the identification of two independent clones containing the light chain 1 (LC1) of MAP1B (Fig. 2b). The interaction between PiT2-loop7 and LC1 in yeast was reconfirmed by co-transformation of LC1 of MAP1B and loop7 of PiT2. The transformants show significant growth on SD/–Ade/–His/–Leu/–Trp selection agar plates, indicating an interaction between LC1 and loop7 (Fig. 2c).

### PiT2 interacts with MAP1B *in vitro* and *in vivo*

**Figure 3:**
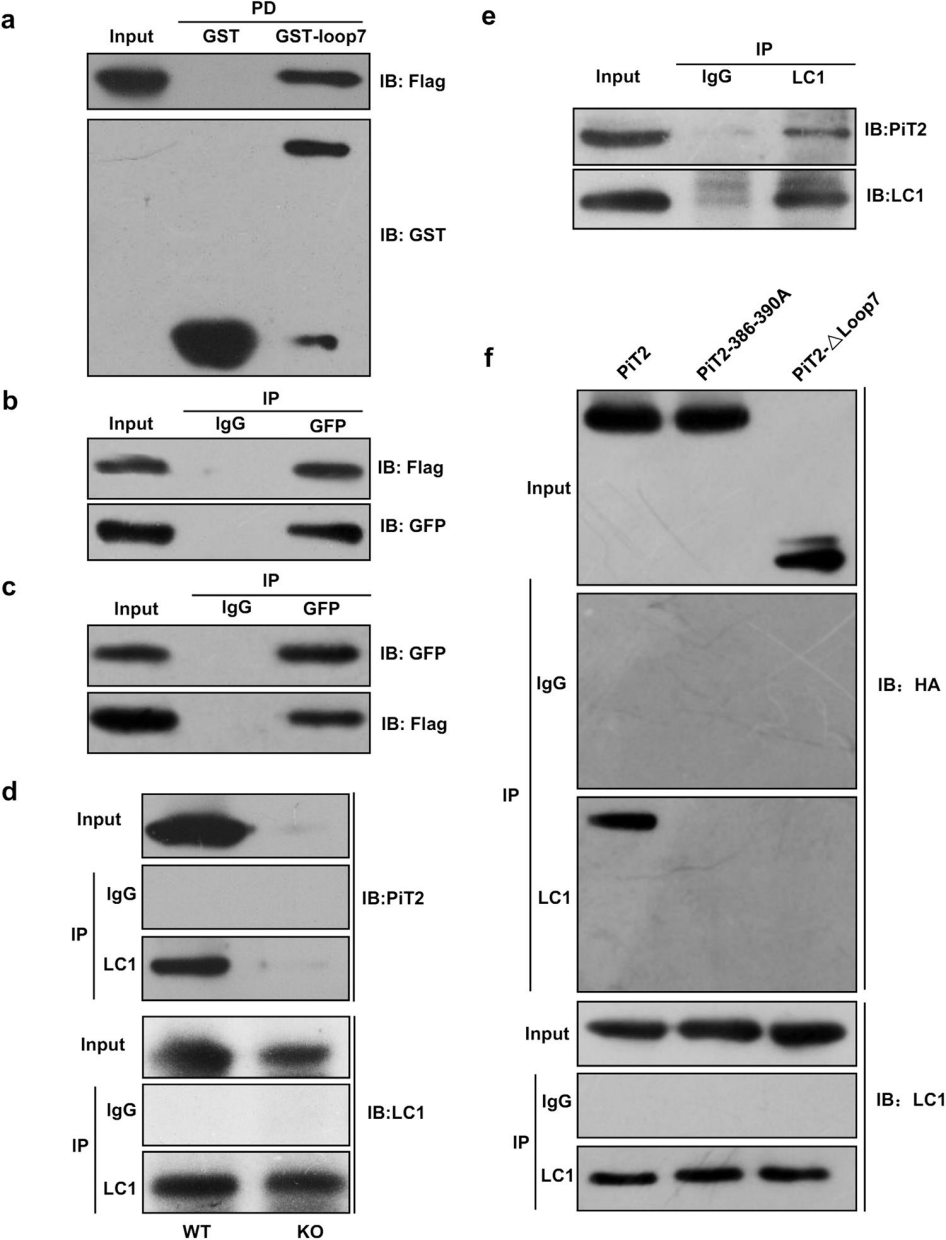
Interaction of PiT2 with MAP1B. (**a**) GST pulldown assays analyzing the interaction between PiT2-loop7 and LC1. Proteins pulled down were detected by using anti-flag antibodies. Full length blots are shown in Supplementary Fig. S3a. (**b**,**c**) Hela cells were co-transfected with PiT2 and LC1 expressing vectors. (**b**) Flag-tagged PiT2 constructs were co-transfected with a GFP-tagged LC1 construct in Hela cells, the GFP-tagged proteins were immunoprecipitated with control IgG or anti-GFP antibodies. Full length blots are shown in Supplementary Fig. S3b. (**c**) Hela cells were co-expressing GFP-tagged PiT2 and flag-tagged LC1, the cell lysates were immunoprecipitated with control IgG or anti-GFP antibodies. The precipitates were immunoblotted with antibodies indicated. Full length blots are shown in Supplementary Fig. S3c. (**d**) Interaction of PiT2 with MAP1B in wild type or *slc20a2* knockout (KO) mice brains. Lysates of mouse brains were immunoprecipitated with LC1 antibody, the precipitates were immunoblotted with anti-PiT2 antibodies. Full length blots are shown in Supplementary Fig. S3d. (**e**) Interaction of PiT2 with MAP1B in Neuro2A cells. Lysates were immunoprecipitated with LC1 antibody, and then blotted with anti-LC1 or anti-PiT2 antibodies. Full length blots are shown in Supplementary Fig. S3e. (**f**) Neuro2A cells were transfected with HA-tagged PiT2-WT, PiT2–386–390A, and PiT2-Δloop7, and the cell lysates were immunoprecipitated with anti-LC1 antibodies. The precipitates were analyzed by immunoblot analysis using the antibodies indicated. Full length blots are shown in Supplementary Fig. S3f.

We substantiated the interaction between loop7 domain and LC1 by GST pulldown assay. The purified GST-PiT2-loop7 fusion protein, instead of GST alone, was able to pull down FLAG-LC1 fusion protein, indicating a direct association between loop7 and LC1 *in vitro* (Fig. 3a and Supplementary Fig. S3a). Then full-length PiT2 and LC1 fusion protein expressing vectors were co-transfected into Hela cells. Lysates from co-transfected cells were immunoprecipitated with GFP antibody. Western blotting showed that GFP antibody was capable of pulling down LC1 and PiT2 fusion protein complexes in Hela cells (Fig. 3b,c and Supplementary Fig. S3b,c). We then carried out co-immunoprecipitation in mouse brain and Neuro2A cells lysates using LC1 antibody followed by Western blotting with PiT2 antibody, the results showed interaction between PiT2 and MAP1B (Fig. 3d,e and Supplementary Fig. S3d,e). After PiT2 knockdown, this interaction was weakened in Neuro2A cells (Supplementary Fig. S4). *In vivo*, no interaction was detected in the supernatant brain lysates of *PiT2* knockout mice (Fig. 3d and Supplementary Fig. S3d).

**Figure 4:**
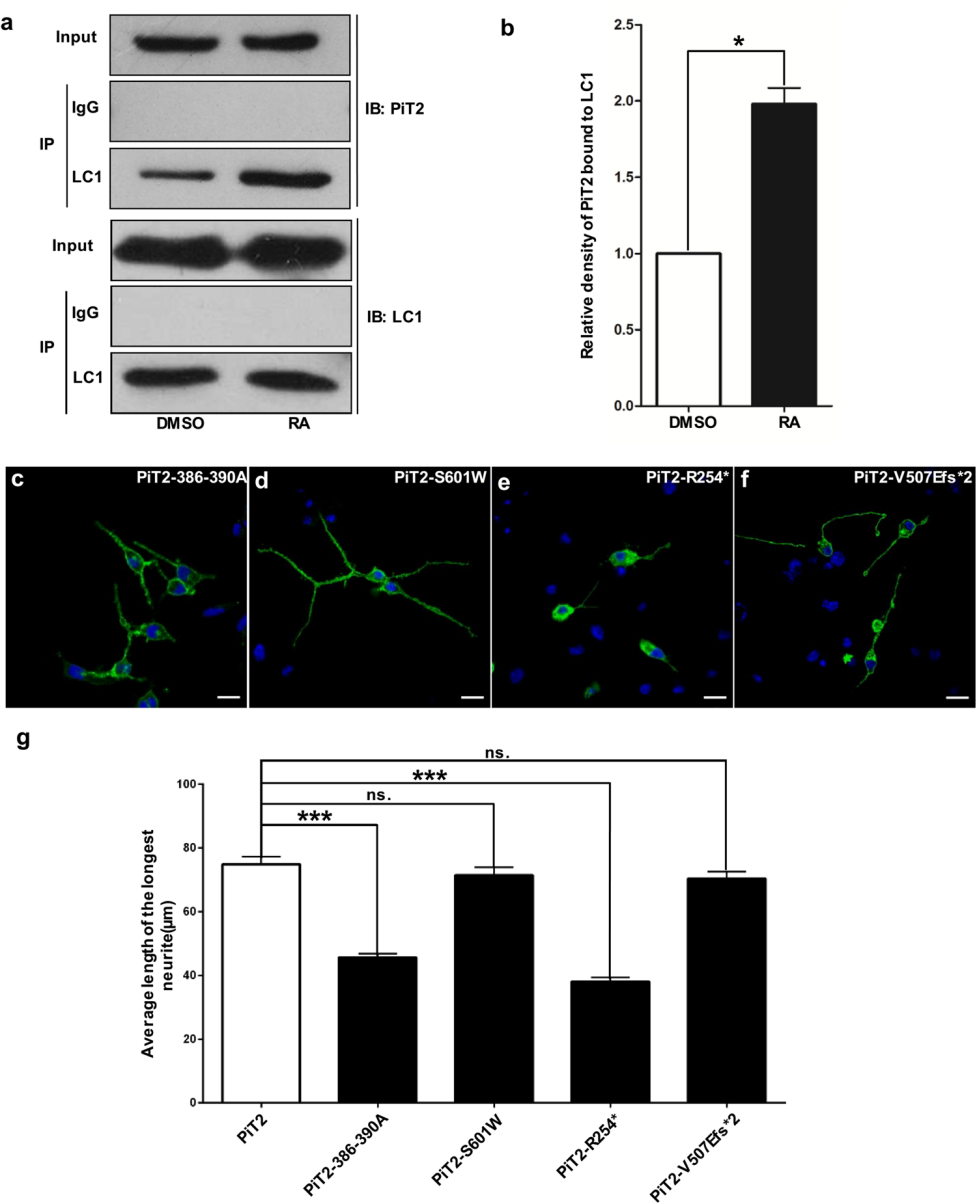
PiT2 with mutated MAP1B binding sites reduces neural outgrowth. (**a**) Co-immunoprecipitation of PiT2 with MAP1B in DMSO- or RA-treated Neuro2A cells. PiT2 interaction with LC1 of MAP1B is significantly enhanced in differentiated cells compared with undifferentiated cells. (**b**) Quantitative analysis (n = 3 independent experiments, * p < 0.05 by Student’s t-test. Data are presented as mean ± s.e.m). Full length blots are shown in Supplementary Fig. S5a. (**c**–**f**) Differentiated Neuro2A cells stained with anti-HA/Alexa Flour488 (green) and DAPI (blue) with overexpression of HA-tagged PiT2–386–390A, PiT2-R254*, PiT2-S601W or PiT2-V507Efs*2 fusion proteins. Scale bar, 20 μm. (**g**) Quantification of neurite length of differentiated Neuro2A cells transfected with the respective plasmids. Bars represent averages of 100 cells from each group in three independent experiments. The error bars represent the mean ± s.e.m. of 100 randomly selected cells from each group in three independent experiments. ns. means no significantly difference; *** means P < 0.001.

MAP1B plays an important role in neurite extension during neuronal differentiation^22^. We performed co-immunoprecipitation in DMSO- or RA-treated Neuro2A cells. Compared with undifferentiated Neuro2A cells, PiT2 proteins co-precipitating with LC1 were roughly doubled in the differentiated Neuro2A cells (Fig. 4a,b and Supplementary Fig. S5a), suggesting that the interaction between PiT2 and MAP1B is affected by the differentiation of Neuro2A cells.

### Mapping and verification of the MAP1B binding site on PiT2

To define the LC1 binding site in loop7 domain, we generated three constructs expressing truncated loop7 domain based on bioinformatics analysis (Supplementary Fig. S6a)^28^. Yeast two-hybrid analysis revealed that deletion of either residues 356–482 or 235–402 prevented binding with LC1, while mutants with deletion of residues 235–355 and 403–482 retained interaction with LC1 (Fig. 2e, Supplementary Fig. S6b). Therefore, we constructed a series of mutants with C-terminal of loop7 truncated and found that only mutants containing residues 235–402 could interact with LC1 (Fig. 2d, Supplementary Fig. S6c). To further localize the binding site of LC1 in PiT2, we generated six overlapping 4-residue alanine substitution mutations in the loop7 domain between residues 384 and 397. Among them, two alanine substitution mutants (residues 386–389 A and 388–390 A) failed to interact with LC1 (Fig. 2f, Supplementary Fig. S6c), which indicate that residues 386–390 (YTCYT) are necessary for interaction between PiT2 and MAP1B.

To further verify the interaction sites, we performed a co-immunoprecipitation experiment in Neuro2A cells. HA epitope tagged PiT2–386–390A mutants were expressed in Neuro2A cells. Western blotting showed that LC1 antibody was able to pull down WT protein, but not 386–390A or Δloop7 mutant proteins (Fig. 3f and Supplementary Fig. S3f).

Previous research results have demonstrated that LC1 is linked to the membrane localization of some ion channels and transmembrane receptor^29–32^. However, by immunofluorescence assay, we found that WT and 386–390A mutant PiT2 proteins localized to plasma membranes, and there were no considerable differences in the fluorescence intensity between WT and 386–390 A mutant (Supplementary Fig. S1a,b).

### Mutating the MAP1B binding site in PiT2 affects neurite length

Next, we conducted immunofluorescence assay and found that overexpressing PiT2 (386–390A) significantly decreased the length of neurites in Neuro2A cells (Fig. 4c,g). This indicates that mutating the MAP1B binding sites in loop7 leads to reduced neurite length.

We also evaluated whether transport function of PiT2 underlies the regulation of neurite outgrowth, and performed neuritogenesis assays in Neuro2A cells transfected with three PiT2 mutants found in IBGC families (S601W, R254*and V507Efs*2)^13,33,34^. The expression levels of 386–391 A, S601W, R254*and V507Efs*2 mutant in Neuro2 cells were similar (Supplementary Fig. S5b-c). Following induction of differentiation, Pi transport function deficient mutant PiT2-S601W (a missense mutation in C-PD domain) and PiT2-V507Efs*2 (deleted C-PD domain only) did not affect the neurite outgrowth in Neuro2A cells (Fig. 4d,f,g). The mutant PiT2-R254* (deleted loop7 and C-PD domain) as similar to PiT2–386–390A and PiT2-Δloop7, leading to significantly decreased length of neurites in Neuro2A cells (Fig. 4e,g). These results demonstrate that loop7 domain of PiT2 may participate in the regulation of neurite outgrowth in Neuro2A cells, while PiT2 lacking Pi transport function exerts no effect on neurite outgrowth.

### The dPiT loop7 domain plays a crucial role in dPiT function *in vivo*

Given that loop7 domain participates in PiT2 trafficking and regulates neurite outgrowth in Neuro2A cells, we examined if similar functions exist in *Drosophila*. Sequence comparison showed that the *Drosophila* genome contains the homolog of human *SLC20A2*, *CG42575*, which encodes dPiT protein. *Drosophila* dPiT is 38% identical and 56% similar to human PiT2 (Supplementary Fig. S7b). We constructed dPiT loss of function mutants *dPiT* ^*21–4*^and *dPiT* ^*15–1*^ by the CRISPR/Cas9 technology (Supplementary Fig. S7). *dPiT* ^*21–4*^ and *dPit* ^*15–1*^ are frame-shift mutants carrying one base pair deletion at 62^th^ and 615^th^ nucleotide of the *dPit* gene, respectively. These mutants produced a truncated 43 and 191 amino acid peptides, respectively, and only 20 and 178 amino acids, respectively, in the C-terminal of this peptide are in common with WT dPiT protein. Heteroallelic or hemizygous mutants of dPiT which carry each of the mutation on one chromosome and the deficiency *Df(3* *L)ED4470* or *Df(3* *L)BSC817* that removes the entire *dPiT* gene on the other, were all embryonic lethal. Ubiquitous and neuronal overexpression of dPiT-GFP in *dPiT* loss of function mutant background by *actin-Gal4* or *elav-Gal4*, respectively, rescued the lethality of loss of function mutant. However, ubiquitous or neuronal overexpression of dPiT-Δloop7-GFP in *dPiT* loss of function background by *actin-Gal4* or *elav-Gal4* could not rescue the embryonic lethality. These results suggest that *dPiT* is an essential gene for *Drosophila* development. The loop7 domain of dPiT is crucial for the function of dPiT.

### Deletion of loop7 domain affects subcellular distribution of dPiT in *Drosophila* neurons

**Figure 5:**
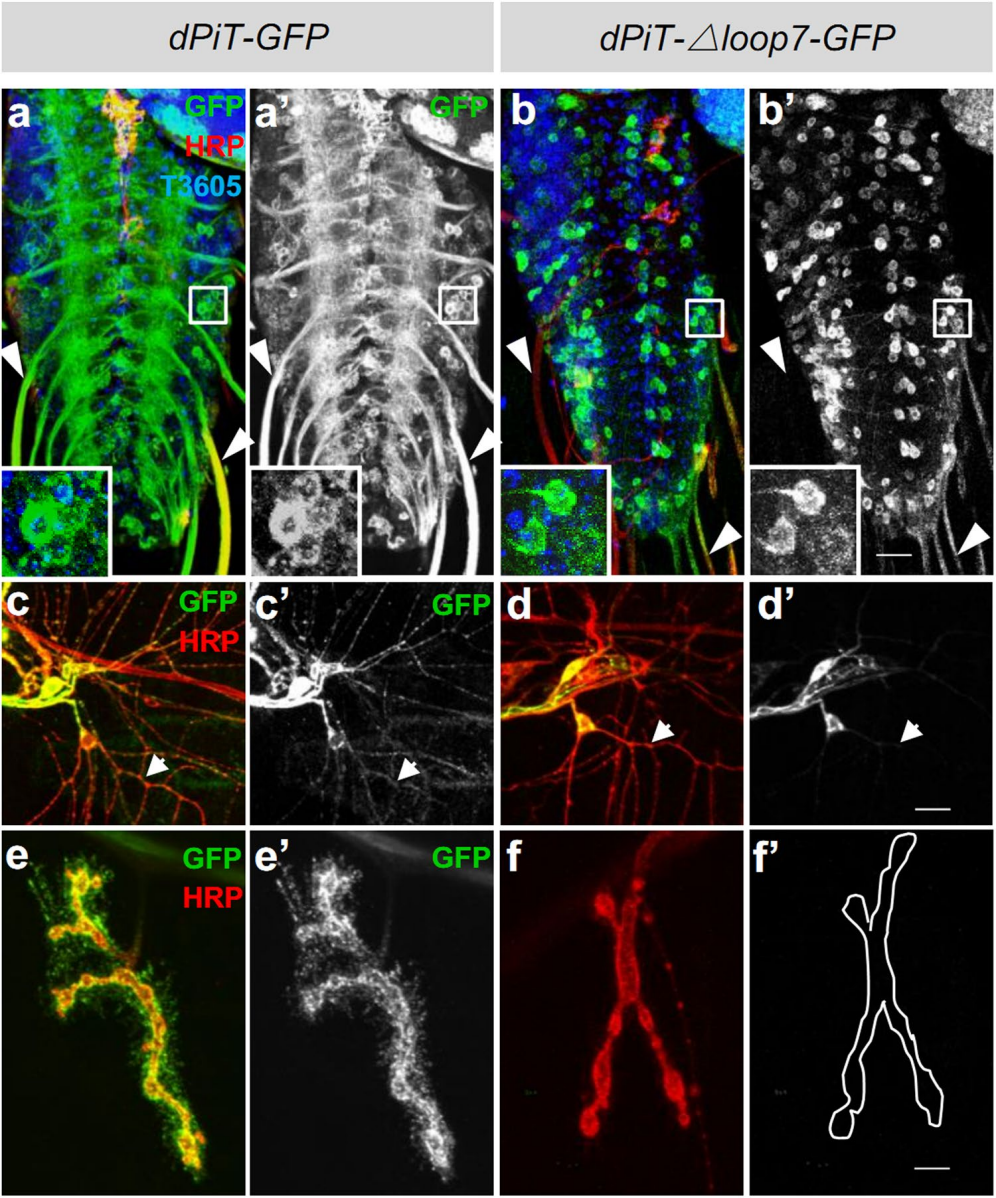
The dPiT loop7 domain plays a crucial role in localization of dPiT. (**a**–f’) The brains from wandering third instar larvae stained with anti-HRP (red), anti-GFP (green) and T3605 (blue) from flies with neuronal overexpression of dPiT-GFP (**a**,a’) or dPiT-Δloop7-GFP (**b**,b’), driven by *elav-Gal4* in wild type background. Scale bar, 10 μm. The dendrites from wandering third instar larvae segment 4 stained with anti-HRP (red), anti-GFP (green) of flies expressing dPiT-GFP (**c**,c’) or dPiT-Δloop7-GFP (**d**,d’) driven by *elav-Gal4* in wild type background. Scale bar: 10 μm. Representative muscle 4 NMJs of the abdominal segment A3 are shown for dPiT-GFP (**e**,e’), dPiT-Δloop7-GFP (**f**,f’) in wild type background. Scale bar, 5 μm. Arrows in **a**-b’ indicate axons. Arrows and arrowheads in c-d’ indicate dendrite branches.

Since aforementioned *in vitro* study showed that the loop7 domain played a crucial role in the trafficking of the PiT2, we then investigated the distribution of dPiT-WT and dPiT-Δloop7 in the neuronal system *in vivo*. Both dPiT-GFP and dPiT-Δloop7-GFP, when driven by *elav-Gal4* in the wild-type background, were abundantly expressed in the cell body of *Drosophila* brain or ventral ganglions. While dPiT-GFP could also be detected in the axon and the terminal of NMJ, there were little distribution of dPiT-Δloop7-GFP in the axon, and it was hardly detectable in the NMJ (Fig. 5a,b’ and 5e,f’). dPiT-GFP was found to be located in all parts of the sensory neuron system, including the cell body and dendrite branches. Nonetheless, dPiT-Δloop7-GFP mainly existed in the cell body and hardly observed in the dendritic branches of *elav-Gal4/*+ *;UAS-dPiT-*Δ*loop7-GFP/*+ flies (Fig. 5c,d’). These results indicate that loop7 domain affects subcellular localization of dPiT in neurons *in vivo*.

### dPiT is required for normal the development of *Drosophila* NMJ synapses

To determine whether dPiT plays a role in synaptic development, we stained larval NMJs with anti-horseradish peroxidase (HRP) to label the neuronal membrane, and anti-cysteine string protein (CSP) to label synaptic vesicles. Knocking down dPiT in neurons or overexpressing dPiT-Δloop7-GFP was found to be associated with striking NMJ abnormalities as compared with controls or flies overexpressing of dPiT-GFP. The efficacy of the RNAi lines was validated by Western blot analysis with dPiT antibody that we generated and reduction of dPiT levels can be observed (Supplementary Fig. S8e).

Comparing with the controls, NMJ length and bouton numbers were significantly decreased when *dPiT* is knocked down in neurons (Supplementary Fig. S8). The length of NMJ 4 from the abdominal segment A3 was significantly decreased from 110.3 ± 3.4 µm in *elav-Gal4/*+ (n = 37) to 98.3 ± 4.3 in *elav-Gal4*/+ ;*UAS-dPiT RNAi* /+ (n = 30, P < 0.05) (Fig. S8a,b,f). The average NMJ length in knockdown dPiT neurons of flies was 89% that of *elav-Gal4*/+ control. We quantified the total number of boutons of NMJ 4 from the abdominal segment A3 and found that knocking down dPiT in neurons significantly decreased bouton numbers. Total number of boutons in the control genotype *elav*-*Gal4*/+ was 22.4 ± 0.7 (n = 40) and 18.6 ± 1.2 in *elav*-*Gal4*/+ ;*UAS-dPiT RNAi*/+ (Supplementary Fig. S8a,b,g). When overexpressing dPiT in neurons with *elav-Gal4*, the NMJ length (114.8 ± 4.6, n = 20, P > 0.05) and bouton number (23.2 ± 0.6, n = 20, P > 0.05) were not significantly different from controls (Supplementary Fig. S8a,e,f,g).

Comparing with genetic control and neuronal overexpression of dPiT-GFP, NMJ length and bouton number were significantly decreased in neuronal overexpression of dPiT-Δloop7-GFP (Supplementary Fig. S8a,c,d,h,i). The length of NMJ 4 from the abdominal segment A3 was significantly decreased from 116.9 ± 3.9 µm in *elav-Gal4*/+ (n = 40, P < 0.001), 123.4 ± 4.7 µm in *UAS-dPiT-GFP*/+ (n = 40, P < 0.001), 108.2 ± 6.0 µm in *UAS-dPiT-Δloop7-GFP*/+ (n = 38, P < 0.05), 107.7 ± 4.5 µm in *elav-Gal4*/+; *UAS-dPiT-GFP*/+ (n = 20, P < 0.01) to 86.3 ± 3.6 µm (n = 22) in *elav*-*Gal4*/+ ;*UAS-dPiT-*Δ*loop7-GFP*/+ flies. The average NMJ length in neuronal overexpression of *dPiT-*Δ*loop7-GFP*/+ flies was 74% of the *elav-Gal4*/+ control (Supplementary Fig. S8h). We quantified the total number of boutons of NMJ 4 from the abdominal segment A3 and found that neuronal overexpression of dPiT-Δloop7-GFP significantly decreased bouton numbers. Total number of boutons in genetics control *elav-Gal4*/+ (23.3 ± 1.0, n = 40, P < 0.001), *UAS-dPiT-GFP*/+ (23.6 ± 0.9, n = 41, P < 0.001), *UAS-dPiT-Δloop7-GFP*/+ (19.6 ± 1.0, n = 38, P < 0.01) decreased to 15.8 ± 0.6 (n = 22) in *elav*-Gal4/+; *UAS-dPiT-*Δ*loop7-GFP*/+ (Supplementary Fig. S8i). However, when overexpressing dPiT-GFP in neurons with *elav-Gal4* (20.1± 1.8, n = 20, P > 0.05), the number of boutons was not significantly different from all controls. Meanwhile, there was significant difference between *elav-Gal4*/+ ;*UAS-dPiT-GFP*/+ and *elav*-*Gal4*/+;*UAS-dPiT-*Δ*loop7-GFP*/+ (P < 0.001) in NMJ bouton number (Supplementary Fig. S8i).

### dPiT regulates NMJ development by interaction with Futsch

**Figure 6:**
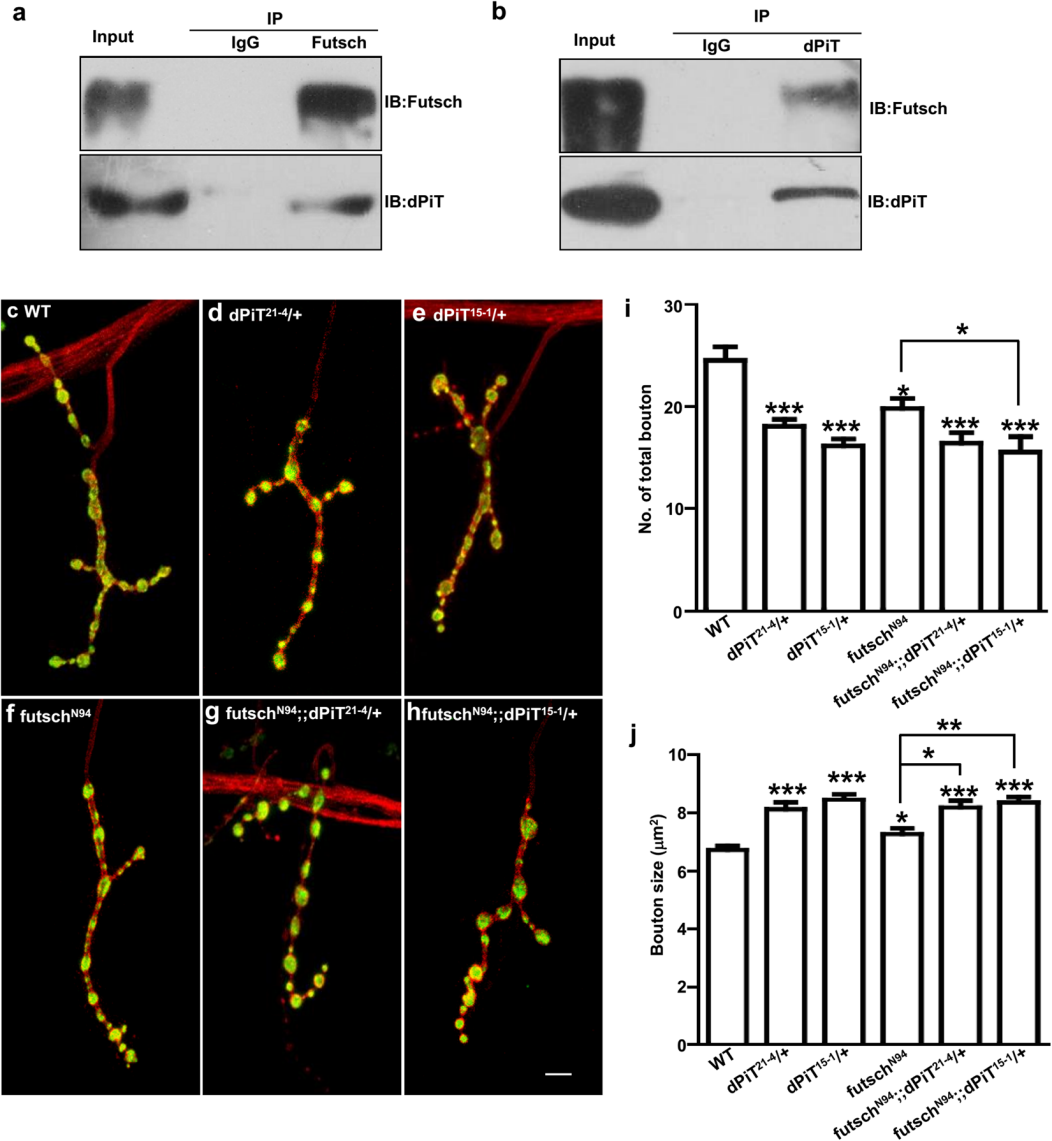
dPiT interacts with Futsch and regulates synaptic growth in *Drosophila*. (**a**,**b**) Co-immunoprecipitation assays analyzing the interaction between dPiT and Futsch in wild type *Drosophila*. Lysates of *Drosophila* brains were immunoprecipitated with anti-Futsch or anti-dPiT antibody. The precipitates were immunoblotted with antibodies indicated. Full length blots are shown in Supplementary Fig. S9. (**c**–**h**) Confocal images of muscle 4 NMJ synapses of abdominal segment A3 double-labeled with anti-HRP (red) and anti-CSP (green). Representative NMJ synapses of different genotypes are shown: WT control (wild type; **c**), *dPiT* mutants *dPiT* ^*21–4*^/+ (**d**) and *dPiT* ^*15–1*^/+ (**e**), *futsch* ^*N94*^ (**f**), *futsch* ^*N94*^; *dPiT* ^*21–4*^/+ (**g**) and *futsch* ^*N94*^; *dPiT* ^*15–1*^/+ (**h**). Scale bar: 5 μm. Quantification of the total number of boutons (**i**) and bouton size (**j**) in different genotypes. Comparisons were made between each genotype and its corresponding control by one-way ANOVA unless indicated otherwise. * Means P < 0.05; ** means P < 0.01; *** means P < 0.001. Error bars indicate s.e.m.

Microtubule-associated protein, Futsch is specifically expressed in *Drosophila* nervous system, and colocalizes with microtubule cytoskeleton in the well-studied *Drosophila* larval NMJ^24,26,35^. To test whether dPiT interacts with Futsch in the central nervous system, we performed immunoprecipitation using *Drosophila* brain. Western blotting of the immunoprecipitates exhibited an interaction between dPiT and Futsch in the brain (Fig. 6a,b and Supplementary Fig. S9).

To investigate the localization pattern of dPiT in *futsch* mutant background, we constructed dPiT::GFP fly that expressed the reporter gene GFP under the *dPiT* control. While the futsch expression level were significantly decreased to 20% of wild type in futsch^N94^ ^24^, the dPiT::GFP intensity was also decreased in axon tracts of ventral nerve cord compared with control, illustrating an effect of Futsch on subcellular localization of dPiT (Supplementary Fig. S10a-b). Meanwhile, comparing with the control, the average dPiT::GFP intensities (6.5 × 10^−4^ ± 0.2 × 10^−4^, n = 3), normalized to corresponding HRP staining of NMJ in control flies was also decreased (Supplementary Fig. S10c-d) to 36.9 × 10^−4^ ± 13.5 × 10^−4^ (n = 6, P < 0.05) in futsch^N94^ mutants (Supplementary Fig. S10e).

*futsch* ^*N94*^ mutant animals have a distinct phenotype with a reduction in bouton number and an enlargement in bouton size (Fig. 6f,i,j)^24^. The *dPiT* mutants show phenotypes in bouton number and bouton size similar to futsch mutants (Fig. 6d,e,i,j). Total number of boutons in wild type (24.5 ± 1.4, n = 18) decreased to 18.1 ± 0.7 (n = 26, P < 0.001) in *dPiT* ^*21–4*^/+ and 16.2 ± 0.7 (n = 25, P < 0.001) in *dPiT* ^*15–1*^/+ (Fig. 6c,d,e,i). The bouton size in wild type 6.73 ± 0.3 μm^2^ (n = 18) increased to 8.1 ± 0.4 μm^2^ (n = 26, P < 0.001) in *dPiT* ^*21–4*^/+ and 8.5 ± 0.3 μm^2^ (n = 25, P < 0.001) in *dPiT* ^*15–1*^/+ (Fig. 6c,d,e,j). We tested for genetic interactions between *dPiT* and *futsch* using double mutants. Bouton number and size pheontypes in *dPiT* mutants on wild-type background is not significantly different from *dPiT* mutants on *futsch* ^*N94*^ background, suggesting that dPiT and Fusch function in a common pathway to regulate bouton growth (Fig. 6). The bouton numbers of *dPiT* ^*21–4*^/+ and *dPiT* ^*15–1*^/+ mutants on *futsch* ^*N94*^ background is 16.4 ± 1.0 (n = 26, P > 0.05) and 15.5 ± 1.5 (n = 25, P > 0.05), comparable with *dPiT* mutants on wild-type background (Fig. 6i). The bouton size of *dPiT* ^*21–4*^ and *dPiT* ^*15–1*^ mutants on *futsch* ^*N94*^ background is 8.2 ± 0.4 µm^2^ (n = 26, P > 0.05) and 8.4 ± 0.4 µm^2^ (n = 26, P > 0.05) has no significantly difference with in *dPiT* mutants on wild-type background (Fig. 6j).

## Discussion

Previous studies and bioinformatics prediction showed that PiT2 is a highly hydrophobic protein consisting of 12 transmembrane domains (TMDs) and a large central intracellular loop (loop7) whose function remains unknown^14,20^. In this study, we found that MAP1B was a new interacting protein of loop7 domain. The interaction between PiT2 and MAP1B was demonstrated by yeast two-hybrid, GST pulldown and co-immunoprecipitation analysis. We found that the interaction was enhanced during the differentiation of Neuro2A cells. Overexpression of PiT2 with mutated MAP1B binding site resulted in a significant decrease in the neurite length of Neuro2A cells compared with wild type. Overexpression of Pi transport function deficient mutants PiT2-S601W and PiT2-V507Efs*2 did not affect neurite outgrowth in Neuro2A cells. These results suggest that PiT2 modulates neurite outgrowth independently of its Pi transport function. *In vivo* studies showed that dPiT possessed similar funtions in *Drosophila*. *Drosophila* dPiT interacts with Futsch, and dPiT is crucial for normal development of *Drosophila* NMJ synapses. Our data support the notion that loop7 domain of PiT2 is implicated in the growth and development of neurons by interacting with the adaptor protein MAP1B.

Most of the PiT2-Δloop7 proteins were localized to a specific region of cytoplasm (Supplementary Fig. S1c). Previous studies have reported that MAP1B can mediate microtubular trafficking of Nav1.6 and 5-HT6R to the cell surface^29,30^. On the other hand, MAP1B interacts with CaV2.2 and 5-HT3A to reduce their expression in the plasma membrane and promoting their desensitization^31,32^. In this study, we found that mutations in residues 386–390 (YTCYT) impeded the interaction between PiT2 and MAP1B but did not affect its localization (Supplementary Fig. S1b). *In vivo* studies also revealed that dPiT-Δloop7-GFP fusion proteins predominantly existed in the cell body but not in axons, the branches of dendrites or the terminal of motor neurons in the *elav-Gal4-*driven *UAS*-*dPiT-*Δ*loop7-GFP* flies (Fig. 5a–f’). Our results demonstrate that loop7 domain is required for membrane localization of PiT2 and interaction between PiT2 and MAP1B, but these two functions depend on different regions of loop7 domain.

MAP1B is highly expressed during early neuronal development^36,37^. MAP1B is principally expressed in neurons, oligodendrocytes, astrocytes, and their progenitor cells^38,39^. The expression pattern of MAP1B is similar to that of PiT2. MAP1B can regulate the dynamic balance between actin and microtubules, and it is essential for axonal growth, branching and nerve regeneration in developing nervous system^40–42^. In this study, we found that knockdown of PiT2 decreased the length of neurites in Neuro2A cells (Fig. 1d,e,g). The interaction between MAP1B and PiT2 was enhanced in differentiated Neuro2A cells (Fig. 4a,b) and abolishing the interaction decreased the length of neuritis (Fig. 4c,g). These findings suggested that the interaction between PiT2 and MAP1B might be involved in the differentiation of Neuro2A cells. Fly embryonic lethality of *dPiT* loss of function mutant illustrated that dPiT is an essential gene for fly development. We checked the NMJ phenotypes in *dPiT* loss of function mutant flies rescued with ubiquitous or neuronal expression of dPiT, respectively, and did not find any significant difference with wild type control in NMJ length and total bouton number (data not shown). This suggests that the crucial role dPiT plays development takes place in the neurons.

Immunoprecipitation assays showed that dPiT formed a complex with Futsch in *Drosophila* brain (Fig. 6a,b and Supplementary Fig. S9). Deletion of loop7 domain prevents dPiT from appropriate subcellular localization and affects normal protein function (Fig. 5). Moreover, *dPiT* and *futsch* mutants exhibit similar bouton growth phenotypes, and the phenotype of double mutants are similar to *dPiT* single mutants. Taken together, our results suggest that PiT2 regulates neuronal growth by interacting with microtubule-related protein MAP1B.

Although Pi uptake of PiT2 in *Xenopus laevis* oocytes showed that loop7 domain is not required for Pi transport function^15^, there might be other regulatory mechanism for Pi uptake in the nervous system. PiT2 is a highly expressed inorganic phosphate transporter in the nervous system^10,43^. PiT2 could interact with actin and change their conformation to adapt to a changing ambient Pi concentration^44–46^. These results indicate that cytoskeletal proteins might play an important role in the regulation of PiT2 transport activity, and this might be related to the interaction between PiT2 and MAP1B in neuronal outgrowth regulation. In this study, we found that Pi transport function deficient mutant PiT2-S601W and PiT2-V507Efs*2 did not affect neurite outgrowth in Neuro2A cells (Fig. 4d,f,g). On the other hand, similar to PiT2-Δloop7, PiT2-R254* which also removes loop7 showed abnormal cytoplasmic localization and significantly decreased length of neurites in Neuro2A cells (Fig. 4e,g). These results show that PiT2 modulates neural outgrowth independently of its Pi transport function.

In summary, we identify a novel function of PiT2, which takes part in the growth and development of nerve cells. Furthermore, we find that PiT2 regulated the differentiation of nerve cells through interaction with MAP1B and independently of its Pi transport function. These findings might provide a novel mechanism that PiT2 regulates neural outgrowth, a process that might contribute to neuronal development.

## Methods

### Yeast Two-hybrid Assay

Yeast two-hybrid experiments were performed using the Matchmaker™ Library Construction & Screening Kits (Clontech Laboratories, Inc., 630445). Briefly, the cDNA sequences encoding the human loop7 domain of PiT2 was amplified from KSM-hPiT2 vector^13^ and subcloned into the pGBKT7 vector for use as “bait” in the yeast two-hybrid screen. A human fetal brain cDNA library as “prey” was purchased from Clontech (Clontech Laboratories, Inc., Mate & Plate™ Library-Human Fetal Brain, 630469). The fetal brain cDNA library was screened by yeast mating, and then the mating mixture was spread onto complete medium lacking leucine, tryptophan, histidine and adenine (SD-Leu/Trp/His/Ade). In order to fully separate AD/library plasmid, candidate clones were restreaked on SD-Leu/Trp/His/Ade medium 2 times, and the β-galactosidase assay was performed using 5-bromo-4-chloro-3-indolyl-β-D-galactopyranoside (Clontech Laboratories, Inc., X-Gal, 8060–1). Plasmid DNA from each yeast colony was isolated and analyzed by polymerase chain reaction (PCR) and sequencing. The library inserts were identified using NCBI-blast search based on the DNA sequence. Bioinformatics analysis of the possible LC1 interaction sites within loop7 of PiT2 were performed using random forest algorithm^28^. Human *MAP1B-LC1* cDNA (NM_005909.4) was amplified from pGADT7-MAP1B (2167–2468) vector (including residues 2167–2468 of MAP1B, which was identified in the screen) (Fig. 2b), and subcloned into pGADT7 vector. The pGBKT7-loop7 construct was used as the parental plasmid to generate the deletion and alanine substitution mutant constructs via PCR mediated mutagenesis^15,47^. The directed tests of the interaction between LC1 and loop7 mutants were performed using LiAc-mediated yeast transformation. The primers are listed in Supplementary Table S1.

### Plasmids and Antibodies

Human *MAP1B-LC1* cDNA was subcloned into p3×flag-CMV-7.1 and pEGFP-N1 vector. The full-length of wild type human *SLC20A2* cDNA (NM_006749) was amplified from KSM-hPiT2 construct and subcloned into pEGFP-N1 vector. Full-length of human *SLC20A2* cDNA with HA epitope tag sequence was subcloned into pCDNA3.1(−) vector, HA tag sequence was introduced into C terminus of PiT2 by PCR using two overlapped reverse primers. The pCDNA3.1-PiT2 construct was used as the parental plasmid to generate the mutant constructs via PCR-mediated deletion or site-directed mutagenesis. The pSIH-shRNA vectors containing either a sequence targeted to the mouse *slc20a2* or a non-silencing control sequence (scramble) were used in RNA interference experiment. The primers are listed in Supplementary Table S2.

For immunodetection, the following antibodies were used at the following dilutions: mouse anti-glutathione S-transferase antibody (ABclonal, AE001; 1:3000 for WB), mouse anti-GFP antibody (Proteintech, 66002–1-Ig; 1:5000 for WB, 1:100 for IP), mouse anti-flag antibody (MBL, M185–3L; 1:5000 for WB, 1:100 for IP), mouse anti-HA antibody (sigma, clone A-7, H3663; 1:2000 for WB), mouse anti-PiT2 antibody (Santa Cruz Biotechnology, sc-101298; 1:200 for WB), rabbit anti-LC1 antibody antibody (Santa Cruz Biotechnology; 1:600 for WB, 1:50 for IP), mouse anti-cysteine string protein antibody [Developmental Studies Hybridoma Bank (DSHB) at the University of Iowa, AB 2307345; 1:500], mouse anti-Futsch antibody (DSHB, AB528403; 1:500) and Texas Red-conjugated goat anti-HRP antibody (1:100; Jackson Laboratory). A rabbit polyclonal anti-dPiT antiserum was raised against the synthetic peptide QSPKEEQKSKTNSIGTD (amino acids 382–398 of dPiT) (Supplementary Fig. S7b).

### Cell culture and transfection

Neuro-2A cells and HeLa cells were respectively cultured in Dulbecco’s modified Eagle medium (DMEM, Thermo Fisher scientific) supplemented with 10% fetal bovine serum (FBS, Thermo Fisher scientific) at 37 °C and in 5% CO_2_. Transiently transfection of cells with plasmid DNA was performed using Lipofectamine® 2000 Transfection Reagent (Thermo Fisher scientific) in Opti-MEM® I Reduced Serum (Thermo Fisher scientific), by following to the manufacturer’s instructions. For induction of differentiation, Neuro2A cells were transiently transfected as mentioned above. 24 h after transfection, the medium was carefully replaced with an equal volume of DMEM with 1% fetal bovine serum and supplemented with 10 μM Retinoic acid (RA) for another 48 h to induce neurite outgrowth.

### Mice

Wild type C57BL/6NTac mice and *Slc20a2* knockout mice C57BL/6NTac-*Slc20a2* ^tm1a-(EUCOMM)Wtsi^/Ieg (European Mouse Mutant Archive. http://www.mousephenotype.org/data/alleles/MGI:97851/tm1a(EUCOMM)Wtsi) were kindly provided by Prof. Xue Zhang (Chinese Academy of Medical Sciences & Peking Union Medical College). Mouse experiments were approved by the Institutional Animal Care and Use Committee (IACUC) at Tonji Medical College, Huazhong University of science and Technology ([2015] IACUC number: 389). All experimental procedures were performed according to relevant guidelines and regulations set by the Tongji IACUC.

### *Drosophila* stocks and husbandry

Flies were cultured on standard cornmeal medium at 25 °C unless otherwise specified. *w* ^1118^ is used as the wild-type control. Other stocks used included the ubiquitous *actin-Gal4* ^48^, muscle-specific *C57-Gal4* ^48^, pan-neuronal *elav-Gal4*, *Df(3* *L)ED4470* and *Df(3* *L)BSC817* which removes *dPiT* completely (Bloomington *Drosophila* Stock Center). *dPiT* RNAi (v49971) line was obtained from Vienna *Drosophila* RNAi Center.

### Generation of UAS transgenic flies

For overexpression studies, a *UAS-dPiT-GFP* construct was made by fusing the *GFP* with the C terminal of *dPiT* (NM_140184.4). Then we transformed *w* ^1118^ *Drosophila* with a *UAS- dPiT-GFP* fusion vector to generate transgenic flies. We also generated the *UAS-dPiT-*Δ*loop7-GFP* vector. The insertion fragment was amplified from *dPiT* cDNA, connecting the sequence that encoding the 239 amino acids of N terminal to that of 183 amino acids of C terminal. Then we fused GFP with the C terminal of dPiT-Δloop7 fragment to generate the *UAS-dPiT-*Δ*loop7-GFP* transgenic flies. The primers are listed in Supplementary Table S3.

### Mutagenesis of *dPiT* by CRISPR/Cas9 System

*Drosophila* Cas9/sgRNA system was used to mutagenize *dPiT* ^49,50^. Two *U6b-sgRNA* plasmids (sgRNA1: gatgccaaaggcgagtacgaagg and sgRNA2: gatcgaaatccggccttgagcgg) were co-injected into nos-Cas9 transgenic blastoderm embryos to induce double-strand break at the first or the second exon of the *dPiT* (Supplementary Fig. S7a). We got two frame shift mutants, *dPiT* ^*21-4*^ and *dPiT* ^*15-1*^, which was induced by sgRNA1 and the sgRNA2 respectively. *dPiT* ^*21-4*^ was the mutation with one bp deletion at the position of 62^th^ in *dPiT* cDNA. *dPiT* ^*15-1*^ also deleted one bp at the position of 535^th^ in *dPiT* cDNA (Supplementary Fig. S7b). The primers are listed in Supplementary Table S3.

### Generation of endogenous dPiT::GFP by CRISPR/Cas9 System

We generated dPiT::GFP flies by CRISPR/Cas9 System^49,50^. We injected *pCDF3-sgRNA-dPiT* vector, which target a double-strand break (DSB) at the carboxyl terminal of dPiT, into syncytial blastoderm-stage embryos^49,50^. The sgRNA sequence was ACATGGGTGGGGCCTAAAGATGG. We also injected a circular double-stranded plasmid containing the GFP-coding sequence flanked by 1.7- and 1.4-kb homology arms from the *dPiT* locus into vasa-cas9 *pCDF3-sgRNA-dPiT* embryos. The donor template is designed to produce an in-frame insertion of *GFP* within the *dPiT* coding region, leading to a dPiT::GFP protein. The flies that expressed endogenous dPiT::GFP were screened by the GFP signal.

### GST pulldown assay

GST tagged PiT2-loop7 fusion proteins were expressed by addition of isopropylthio-β-galactoside (IPTG, 0.1 mM, Sigma) in *E*.*coli* strain BL21 at 30 °C for 4 h. The HeLa cells were transfected with p3×flag-LC1 voter. Then glutathione-sepharose beads (Merck Millipore) were used to purify the GST fusion proteins according to the manufacturer’s protocol and subsequently incubated with the HeLa cells lysates at 4 °C over night. The pulldown proteins bound to the beads were detected by Western blotting.

### Immunoprecipitation

Expression vectors were transfected into Neuro2A cells using Lipofectamine 2000 (Thermo Fisher scientific). After culturing for 36 h, cells were lysed using IP lysis buffer (Beyotime) supplemented with cock-tail protease inhibitors (Roche). To detect endogenous interaction, 150 *Drosophila* (half male and half female adult flies emerging from the pupal cases within a week) heads or one newborn mouse brain was lysed using IP lysis buffer supplied with the Complete Protease Inhibitor Cocktail (Roche). Cell or tissue lysates were collected, and then centrifuged at 12,000 rpm, 4 °C for 10 min. Supernatants were immuno-precipitated with appropriate primary antibody over night at 4 °C. Then protein A-agarose beads (Merck Millipore) were added and incubated with the samples for another 2 h. For immunoprecipitation of *Drosophila*, lysates were incubated with appropriate primary antibody and Dynabeads® Protein G (Thermo Fisher scientific). The beads were washed with IP lysis buffer three times. The immunoprecipitates were analyzed by Western blotting.

### Western Blotting

Lysates or immunoprecipitates were prepared using SDS sample buffer. Proteins were separated by 10% SDS-PAGE and transferred to nitrocellulose membranes. The membranes were blocked for 2 h at room temperature (RT) with 5% skim milk, and incubated with the appropriate primary antibodies overnight at 4 °C. Next day the membranes were washed three times with TBST buffer (20 mM Tris-HCl, 150 mM NaCl, 0.05% Tween-20, pH7.6), and incubated in HRP-conjugated secondary antibodies (1:20000, goat anti-rabbit or goat anti-mouse, Thermo Fisher scientific) for 2 h at RT. After rinsing three times, the proteins were detected by using SuperSignal ELISA Femto Maximum Sensitivity Substrate (Thermo Fisher scientific). The protein bands were quantitatively analyzed by employing the Image J software package (http://imagej.nih.gov/ij/).

### Immunofluorescence and Microscopic Analysis

For RNA interference experiment, Neuro2A cells were transfected with pSIH-H1-copGFP shRNA vectors, containing either a sequence targeted to PiT2 gene (shRNA-PiT2) or a non-silencing sequence (scramble). Transfected cells were fixed in 4% paraformaldehyde for 10 min and permeabilized with 0.5% Triton X-100 for 2 min at RT. After staining with DAPI (1:1,000, Sigma) for 5 min, cells were preserved at 4 °C. For exogenous expression experiment, transfected cells were fixed in 4% formaldehyde for 10 min at RT and permeabilized with 0.5% Triton X-100 for 2 min. Cells were incubated with the primary antibody overnight at 4 °C. The next day cells were incubated with the secondary antibody (Alexa Fluor 488-conjugated donkey anti-mouse IgG secondary antibody and Alexa Fluor 594 F(ab’)2 fragment of goat anti-rabbit IgG(H+L) secondary antibody, 1:500, Thermo Fisher scientific) at 30 °C for 1 h. After staining with DAPI for 5 min, cells were preserved at 4 °C. For Immunochemical analysis of NMJ, third instar larvae were dissected in HL3 with all internal organs removed, followed by fixation in the 4% paraformaldehyde for 40 min. Dissection and antibody staining of third instar larvae were performed as previously described^51,52^. Immunofluorescent imaging was performed using an Olympus FluoView 1000 Laser Scanning Confocal Microscope mounted on an Olympus IX-81 inverted microscope. Image analysis was carried out using FV1000 Viewer and the Image J software. The length of the longest neurite in each Neuro2A cell stained green or NMJ length of *Drosophila* was measured from the swell of HRP staining to the terminal using Image J software package. All branches were calculated. For bouton size analyses, ImageJ 3.0 (NIH) was used to define anti-HRP-stained individual boutons. The software output reports the area for each bouton automatically. At least 16 NMJ4 terminals of different genotypes were analyzed. For quantification of GFP intensities in NMJ, staining signals were digitalized automatically using ImageJ and normalized to the average intensities in the corresponding HRP staining. 100 cells from each group in three independent experiments were captured for neurite outgrowth assay of Neuro2A cells.

### Statistical analysis

Experiments were repeated at least three times. Statistical analyses were performed with T-test for the comparison of two groups, and one-way ANOVA for the comparison of three or more groups. Quantitative data were presented as mean ± s.e.m., p values < 0.05 were considered to be statistically significant.

## Electronic supplementary material

Supplementary Information
