# Supplementary material for: PiT2 regulates neuronal outgrowth through interaction with microtubule-associated protein 1B
Source: Sci Rep. 2017 Dec 19;7:17850. doi: 10.1038/s41598-017-17953-3 (PMC5736545; doi:10.1038/s41598-017-17953-3)
Supplement: Supplementary file 1 — Supplementary Information [file 41598_2017_17953_MOESM1_ESM.doc]

**PiT2** **regulates** **neuronal outgrowth through interaction with microtubule-associated protein 1B**

Xi-Xiang Ma^1+^, Xiangyang Li^1+^, Ping Yi^1+^, Cheng Wang^1^, Jun Weng^1^, Li Zhang^2^, Xuan Xu^1^, Hao Sun^1^, Shenglei Feng^1^, Kai Liu^1^, Rui Chen^1^, Shiyue Du^1^, Xiao Mao^3^, Xiaomei Zeng^1^, Luo-Ying Zhang^1^, Mugen Liu^1^, Bei-Sha Tang^3^, Xiaojuan Zhu^4^, Shan Jin^2^* & Jing-Yu Liu^1^*

1. Key Laboratory of Molecular Biophysics of the Ministry of Education, Center for Human Genome Research, College of Life Science and Technology, Huazhong University of Science and Technology (HUST), Wuhan 430074*,* China.

2. College of Life Sciences, Hubei Collaborative Innovation Center for Green Transformation of Bio-Resources, Hubei University, Wuhan 430062*,* China.

3. Department of Neurology, Xiangya Hospital, Central South University, Changsha, Hunan 410008, China.

4. College of Life Sciences, Northeast Normal University, Changchun 130024*,* China.

^+^ These authors contributed equally to this work.

*Correspondence and requests for materials should be addressed to J.-Y. L. (liujy@hust.edu.cn) or S. J. (jinshan@hubu.edu.cn)


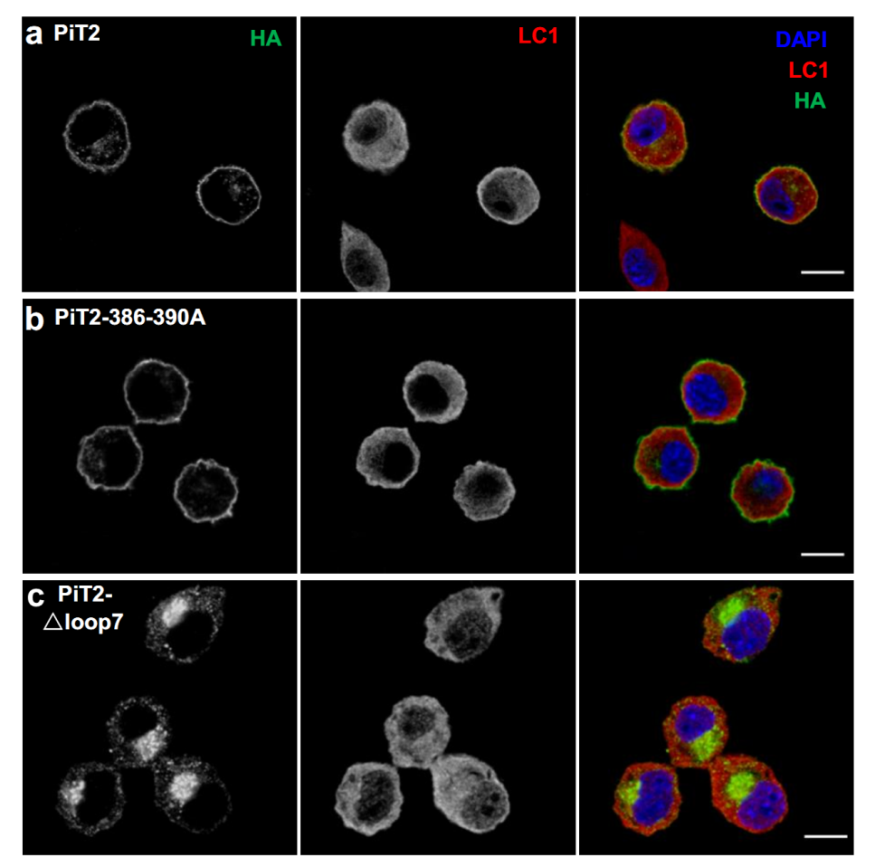


**Supplementary Figure S1. Subcellular localization of wild type PiT2, 386-390A and △loop7 mutants** **in Neuro-2A cells.** (a-c) Undifferentiated Neuro2A cells stained with anti-HA/Alexa Flour488 (green), anti-LC1/Alexa Flour 594 (red), and DAPI (blue) with transiently transfected with HA-tagged PiT2. The yellow signals surrounding the membrane indicate co-localization of PiT2 and LC1. (a, b) HA tagged wild type PiT2 or PiT2-386-390A proteins were co-localized with endogenous LC1 in the membrane region. (c) Most of PiT2-△loop7 proteins were found in a specific region of cytoplasm. Scale bar, 10 μm.


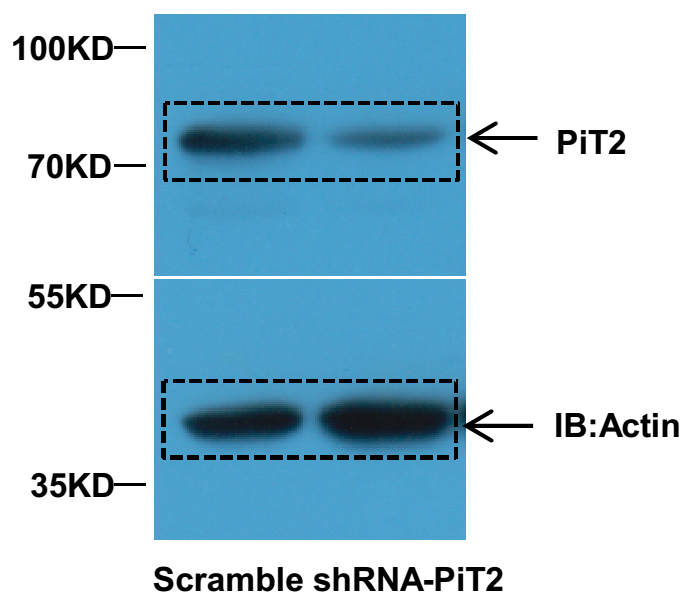


**Supplementary Figure S2. Full length blots for** **Figure 1c*.***

The dotted boxes highlight the cropped areas shown in Figure 1c.


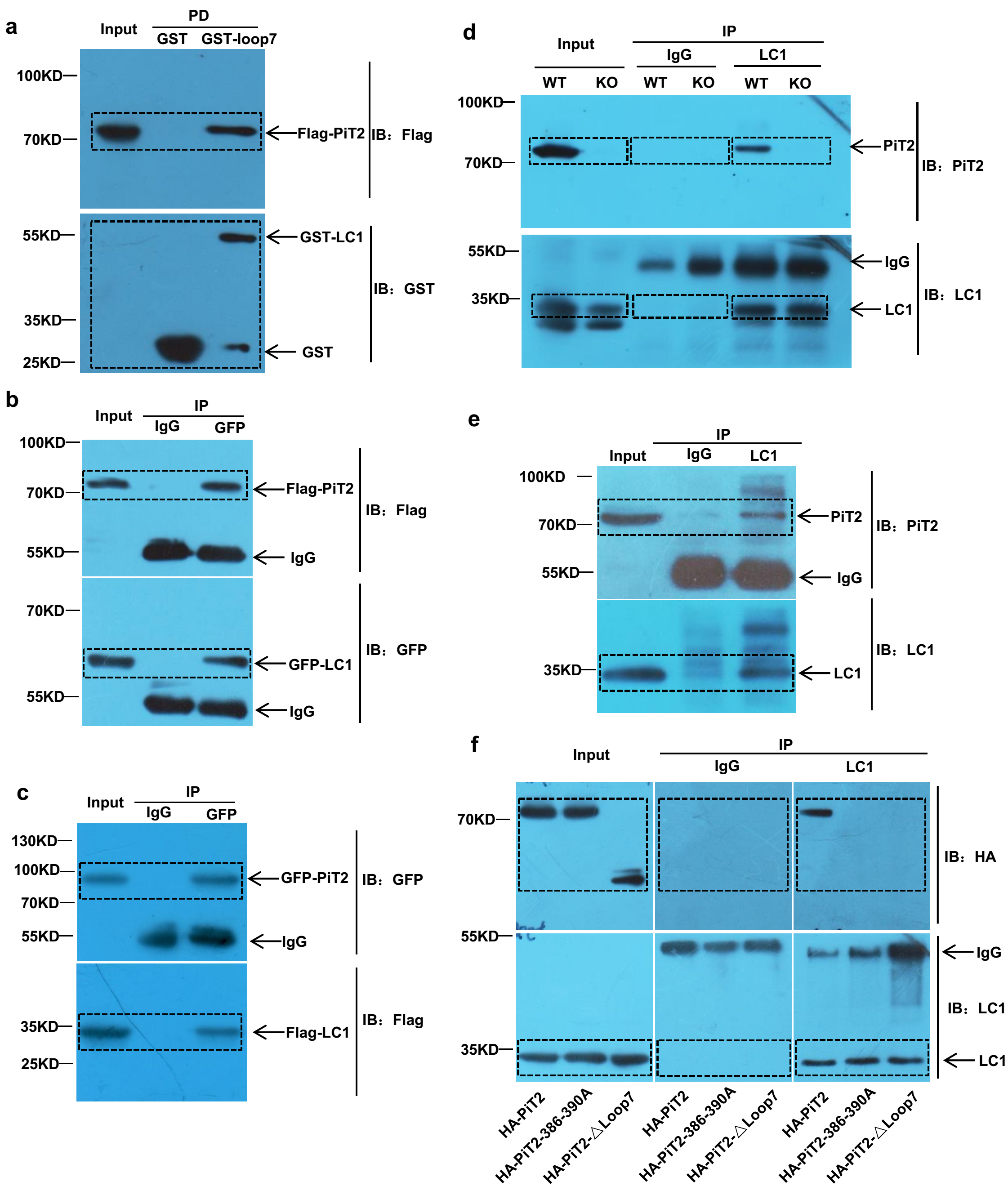


**Supplementary Figure S3. Full length blots for Fig. 3.** Full blot images presented in Figure 3a (a), Figure 3b (b), Figure 3c (c), Figure 3d (d), Figure 3e (e), and Figure 3f (f). The dotted boxes highlight the cropped areas shown in Figure 3.


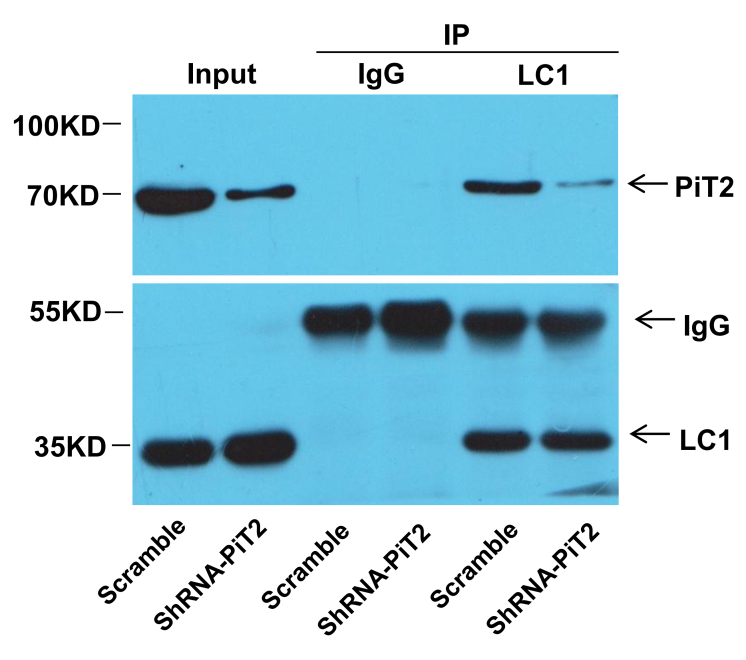


**Supplementary Figure S4.** The interaction of PiT2 with MAP1B in Neuro2A cells with transfection of pSIH-PiT2. Because of shRNA knockdown cells also had a little expression of PiT2, LC1 antibodies could recruit rarely PiT2 protein.


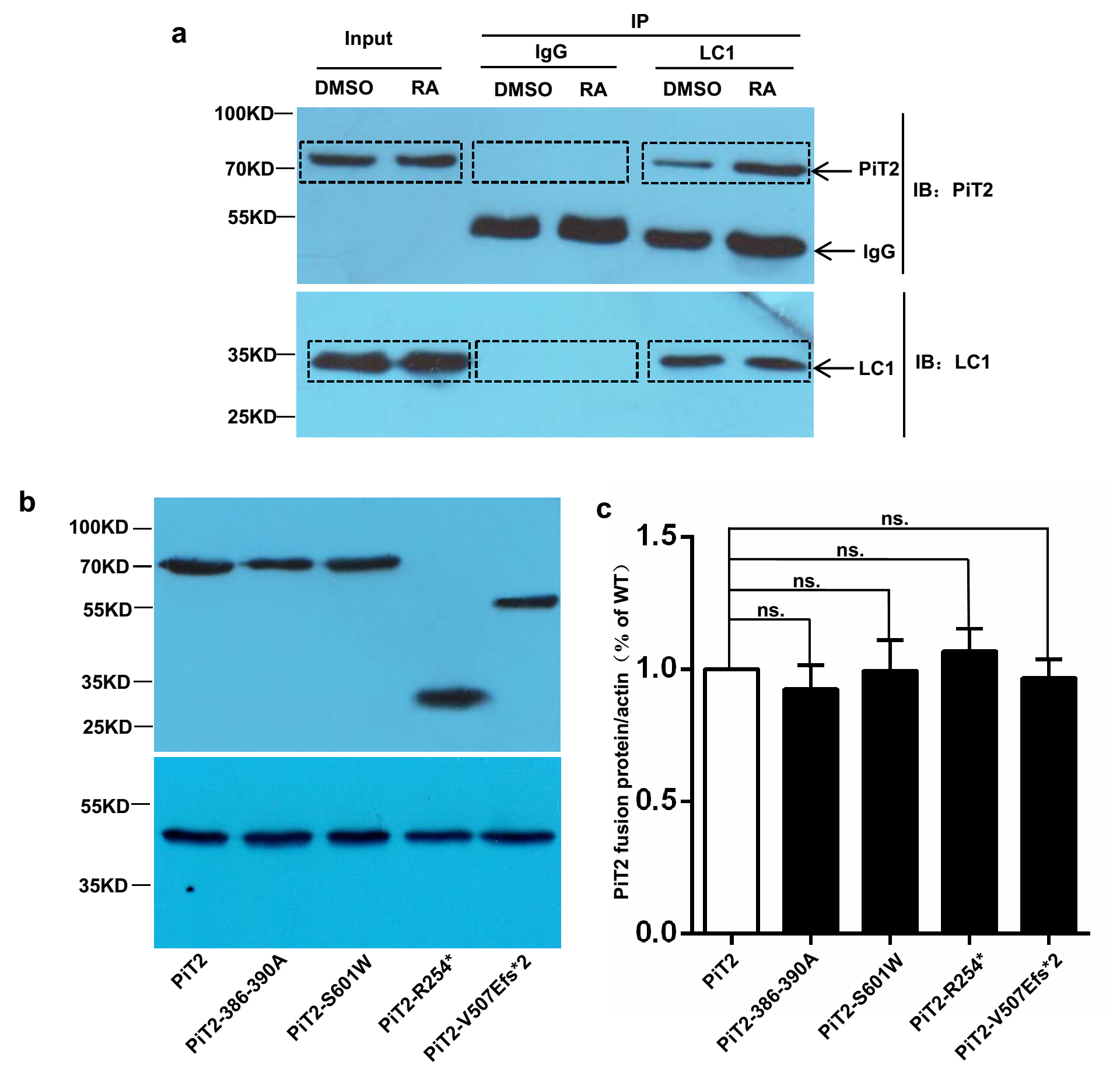


**Supplementary Figure S5.** (a) The dotted boxes highlight the cropped areas shown in Figure 4a. (b) Western blotting analysis of the expression levels of 386-391A, S601W, R254*and V507Efs*2 mutant in Neuro2 cells.


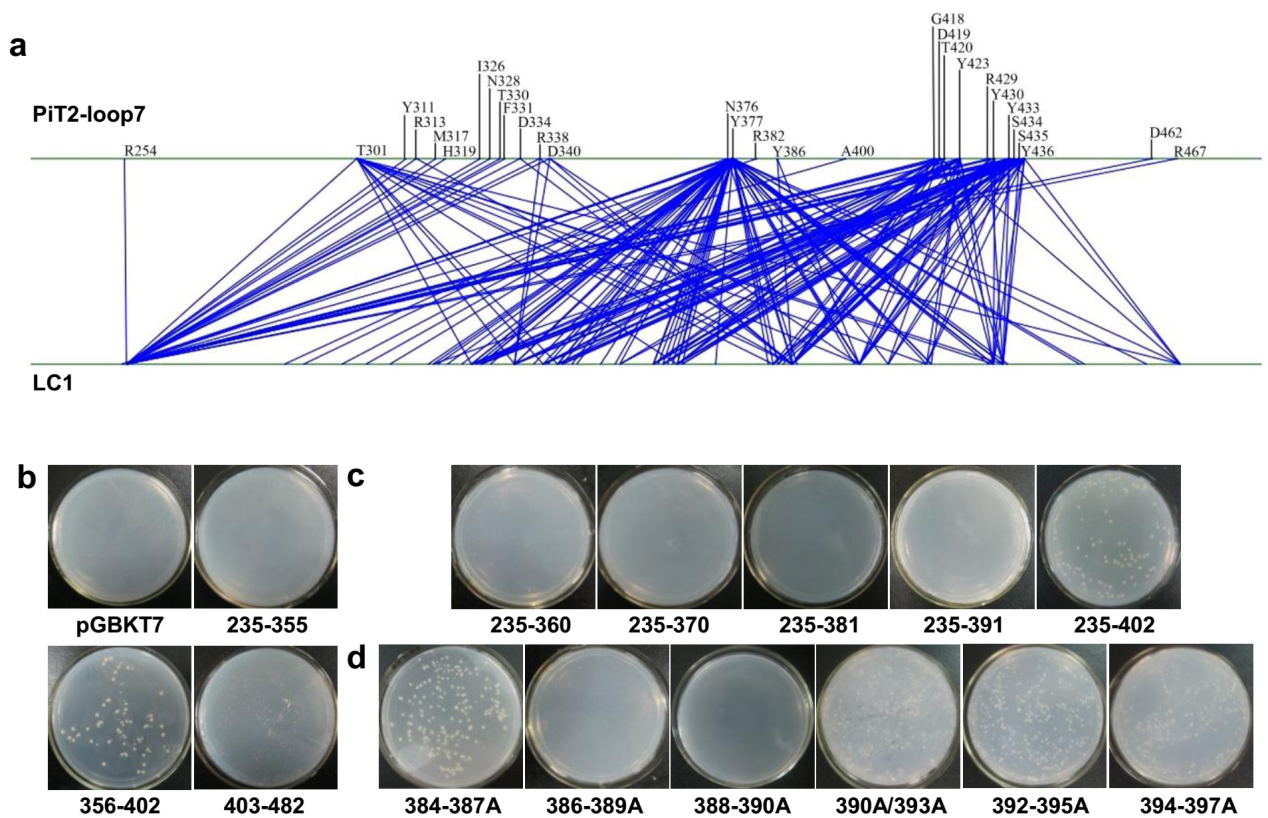


**Supplementary Figure S6. MAP1B binding site in loop7 of PiT2.** (a) Bioinformatic analysis of the possible MAP1B interaction site within loop7 of PiT2. Residues shown in the schematic diagram represent the matthews’s correlation coefficient (MCC) was greater than 2.3. (b) Yeast two-hybrid interaction of LC1 with three deletion mutants of loop7. Only residues 356-402 interacted with LC1. (c) Yeast two-hybrid interaction of LC1 with five C terminal deletion mutants of loop7. Only residues 235-402 interacted with LC1. (d) Yeast two-hybrid interaction of LC1 with six alanine substitution mutants of loop7. Mutations of residues 386-389 and 388-390 impeded the interaction between PiT2 and LC1.


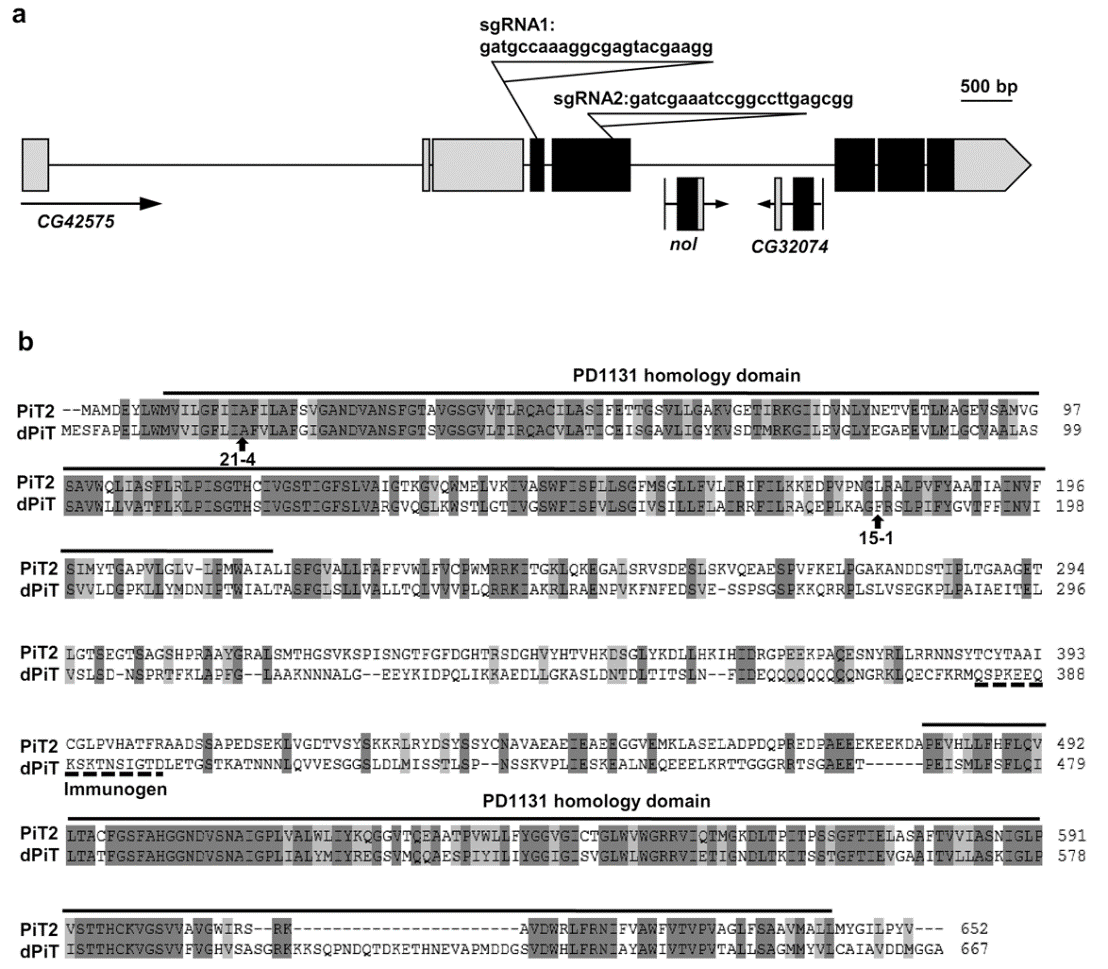


**Supplementary Figure S7. *Drosophila CG42575.*** (a) Genomic structure of *Drosophila* CG42575 and mapping of sgRNA. The intron-exon organization of CG42575, *nol* and *CG32074* were shown in the Schematic. Black boxes, coding regions; Gray boxes, untranslated regulatory regions; ‘gaps’, introns; horizontal line, intergenic region. (b) Sequence alignment of *Drosophila* dPiT and human PiT2. Dark and light-gray shades indicated identical or similar amino acids, respectively. Immunogen of anti-dPiT antibody was indicated by the dotted line.


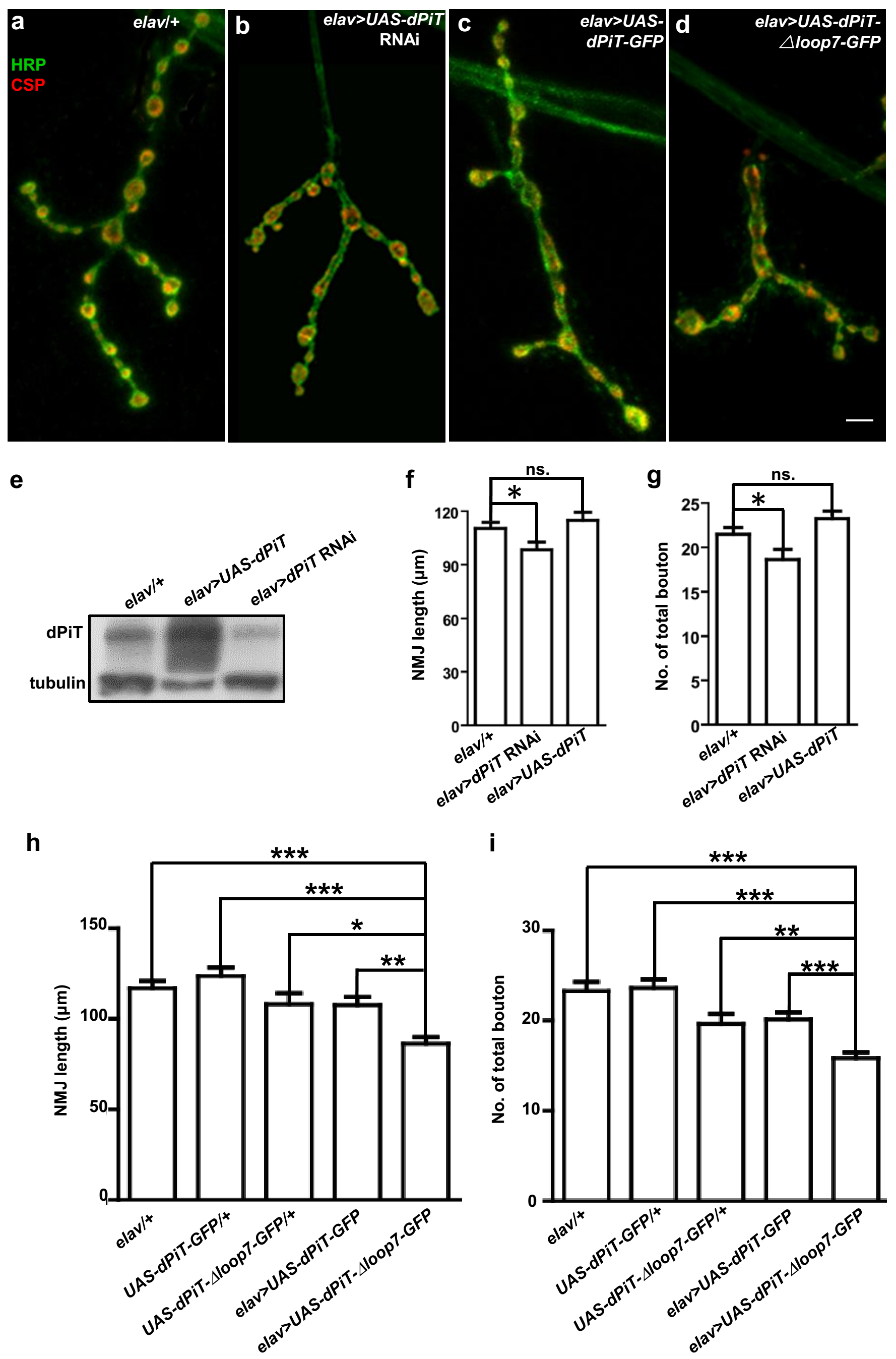


**Supplementary Figure S8.** **dPiT regulates NMJ synapse development.** NMJs from wandering third instar *Drosophila* larvae were stained using anti-HRP (green) and anti-CSP (red) antibodies, respectively. Representative images of the NMJ on muscle 4 of abdominal segment A3 are shown. (a) control genotype *elav-Gal4*/+, (b) *elav-Gal4*/+;*UAS-dPiT RNAi* /+, (c) *elav-Gal4/+*;;*UAS-dPiT-GFP/+* and (d) elav-*Gal4*/+;*UAS-dPiT-Δloop7-GFP*/+. Scale bar: 5 μm. (e) Western analysis of dPiT overexpression and RNAi knockdown transgenic flies. (f-i) Quantification of NMJ length (f, h), bouton number (g, i) for the different genotypes. *P<0.05, **P<0.01, ***P<0.001; error bars indicate s.e.m.


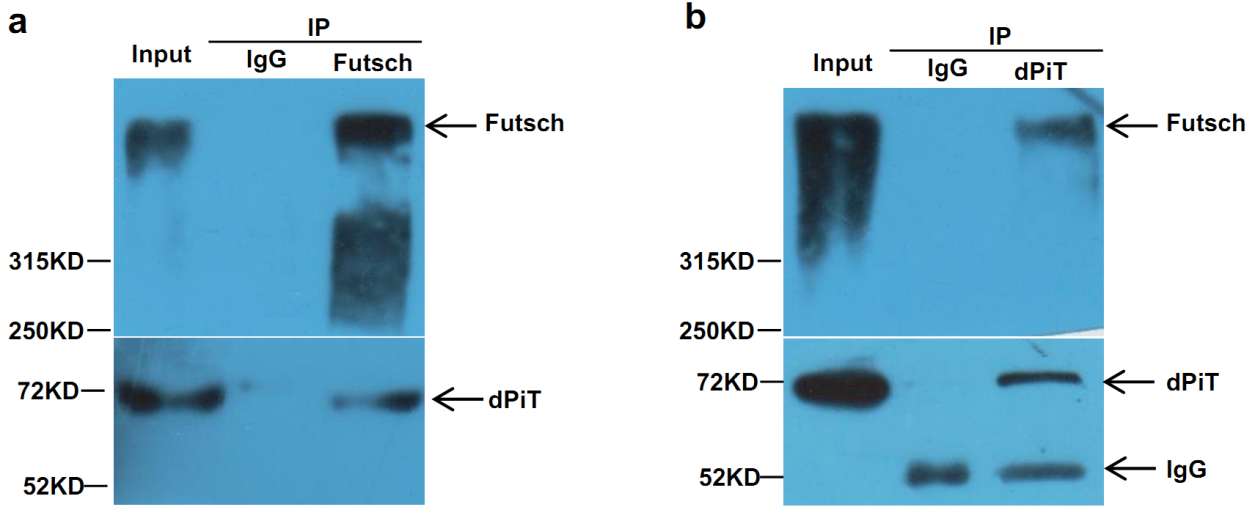


**Supplementary Figure S9. Full length blots for Figure 6a and 6b.**

**
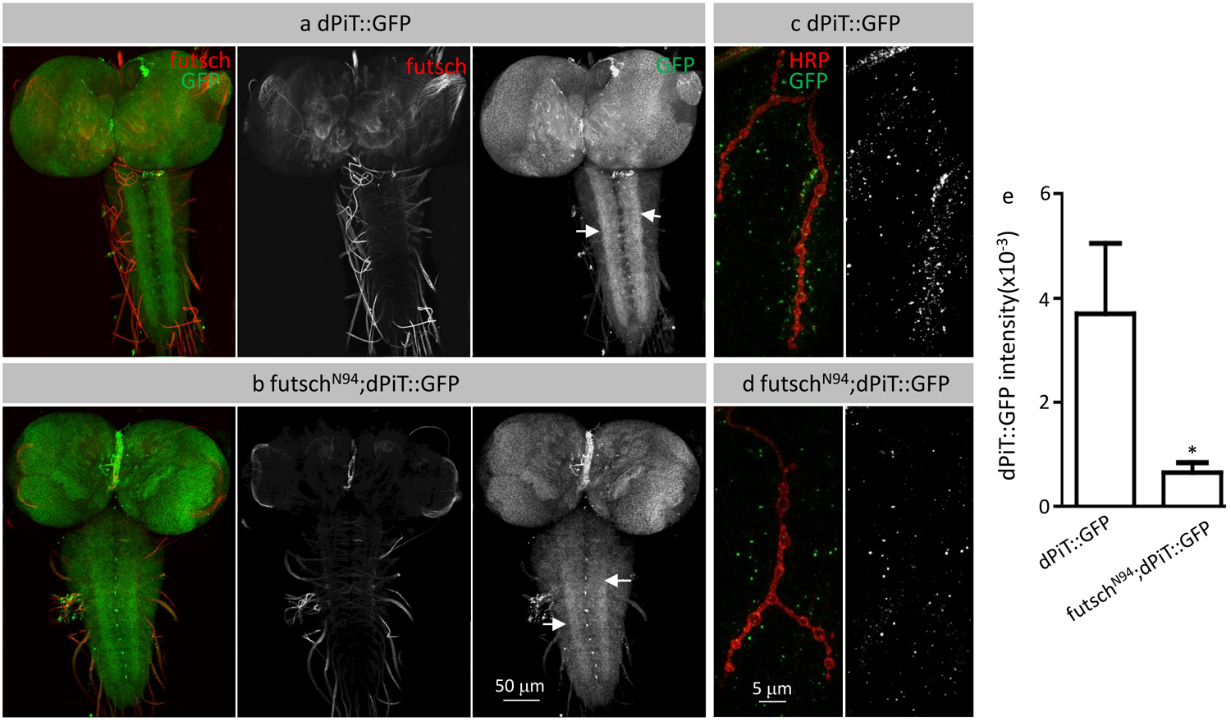
**

**Supplementary Figure S10. Futsch regulated dPiT localization.**

(a) dPiT::GFP control and (b) dPiT::GFP in *futsch*^N94^ mutant background stained with futsch (red) and GFP (green) antibodies. Arrows indicate the ventral nerve cord. (c) dPiT::GFP control and (d) dPiT::GFP in *futsch*^N94^ mutant background stained with HRP (red) and GFP (green) antibody.(e) Statistical results of the GFP staining intensity in NMJ.

**Supplementary Table S1. Primer sequences for yeast two-hybrid**

| Name | Sequence (5′ to 3′) |
| --- | --- |
| pGBKT7-loop7    pGBKT7-loop7(235-355)  pGBKT7-loop7(356-402)  pGBKT7-loop7(403-482)  pGBKT7-loop7(235-391)  pGBKT7-loop7(235-381)  pGBKT7-loop7(235-370)  pGBKT7-loop7(235-360)  pGBKT7-loop7(384-387A)  pGBKT7-loop7(386-389A)  pGBKT7-loop7(388-391A)  pGBKT7-loop7(390-393A)  pGBKT7-loop7(392-395A)  pGBKT7-loop7(394-397A)  pGADT7-LC1 | F: CCGGAATTCTGTCCGTGGATGCGGAGG  R: CGCGGATCCTACTCGGGTGCGTCCTTCTCC  F: CCGGAATTCTGTCCGTGGATGCGGAGG  R: CGCGGATCCTAGTAGAGCCCCGAGTCTTTGTG  F: CCGGAATTCAAAGATCTGCTGCACAAAATCCACA  R: CGCGGATCCTATCGAAAGGTGGCGTGCACTG  F: CCGGAATTCCGCTGCGGACTCATCGGCCC  R: CGCGGATCCTACTCGGGTGCGTCCTTCTCC  R: CGCGGATCCTATGCGGTGTAGCAGGTGTA  R: CGCGGATCCTAGCGCAGCAGCCGGTAGTT  R: CGCGGATCCTACTCCTCGGGGCCCCTGTC  R: CGCGGATCCTATTTGTGCAGCAGATCTTT  F:CGAAACGCAGCAGCAGCATGCTACACCGCAGCCATTTG R:GTAGCATGCTGCTGCTGCGTTTCGGCGCAGCAGCCGGTA  F: AACAGTGCAGCAGCAGCAACCGCAGCCATTTGTGGG  R:TGCGGTTGCTGCTGCTGCACTGTTGTTTCGGCGCAGCA  F: TACACCGCAGCAGCAGCAGCCATTTGTGGGCTGCCAG  R: AATGGCTGCTGCTGCTGCGGTGTAACTGTTGTTTCG  F: TGCTACGCAGCAGCAGCATGTGGGCTGCCAGTGCA  R: CCCACATGCTGCTGCTGCGTAGCAGGTGTAACTGTTG  F:ACCGCAGCAGCAGCAGCACTGCCAGTGCACGCCACCT  R: TGGCAGTGCTGCTGCTGCTGCGGTGTAGCAGGTGTA  F: GCCATTGCAGCAGCAGCAGTGCACGCCACCTTTCGAG  R:GTGCACTGCTGCTGCTGCAATGGCTGCGGTGTAGCAGG  F: CGGAATTCCCACGCCACCCTGATGTGTC  R: CGGGATCCCAGTTCAATCTTGCATGCAG |

**Supplementary Table S2. Primer sequences for GST pull-down assay, Immunoprecipitation and Immunofluorescence**

| Name | Sequence (5′ to 3′) | |
| --- | --- | --- |
| pGEX-6P-1-loop7  pCDNA3.1(-)-PiT2    p3×flag-PiT2  pEGFP-N1-PiT2  p3×flag-LC1  pEGFP-N1-LC1  pCDNA3.1(-)-PiT2-386-390A  pCDNA3.1(-)-PiT2-△loop7  pCDNA3.1(-)-PiT2-S601W  pCDNA3.1(-)-PiT2-R254*  pCDNA3.1(-)-PiT2-V507Efs*  PiT2 shRNA | | F:CGCGGATCC TGTCCGTGGATGCGGAGG  R:CCGGAATTCTCACTCGGGTGCGTCCTTCTCC  F:CCGGAATTCGCAATGGCCATGGCCATGGATGAGTATTTG  R1:CTGGAACATCGTATGGGTACACATATGGAAGGATCCCA  R2:CCCAAGCTTTTAAGCGTAATCTGGAACATCGTATGGGT  F:CCCAAGCTTATGGCCATGGCCATGGATGAGTAT  R: CCGGAATTCACATATGGAAGGATCCCA  F: CCCAAGCTTATGGCCATGGCCATGGATGAGTAT  R: TCCCCGCGGCACATATGGAAGGATCCCA  F: CCCAAGCTTCCACGCCACCCTGATGTGTC  R: CGCGGATCCTTACAGTTCAATCTTGCATGCAG  F: CCGGA ATTCAACCACGCCACCCTGATGTGTC  R: CGCGGATCCCGCAGTTCAATCTTGCATGCAG  F:AACAGTGCAGCAGCAGCAGCAGCCGCCATTTGTGGGCTGCCAG R:AATGGCGGCTGCTGCTGCTGCTGCACTGTTGTTTCGGCGCAGCA  F:GAAGGTGCTTTATCACACCTCCTGTTCCATTTCCTG  R:ATGGAACAGGAGGTGTGATAAAGCACCTTCTTTTT  F: ACTGTAAGGTGGGCTGGGTGGTGGCCGTGGG  R: CCCACGGCCACCACCCAGCCCACCTTACAGT  F: GAAGGTGCTTTATCATGAGTATCTGACGAAA  R: TTTCGTCAGATACTCATGATAAAGCACCTTC  F: ACGGCGGCAATGACGAGTAATGCCATCGGT  R: ACCGATGGCATTACTCGTCATTGCCGCCGT  sense: GATCCCCACAGCTCATCTTCCAGAATCTTCCTGTCAGAA TTCTGGAAGATGAGCTGTGGTTTTTG  antisense: AATTCAAAAACCACAGCTCATCTTCCAGAATTCTGACAGGAAGATTCTGGAAGATGAGCTGTGGG |

**Supplementary S3. Primer sequences for overexpression, null mutant in *Drosophila***

| Name | Sequence (5′ to 3′) |
| --- | --- |
| pUAST-dPiT-GFP pUAST-dPiT-GFP-△loop7  pUAST-dPiT-△loop7  21-4 sgRNA1  15-1 sgRNA2 | F: CTCGAGATGGTGAGCAAGGGCGAGG  R: TCTAGATTACTTGTACAGCTCGTCCATGCC  F1: AGATCTATGGAGAGCTTTGCGCCGG  R1: CTCGAGGGCCCCACCCATGTCGTCC  F2: CTCGAGATGGTGAGCAAGGGCGAGG  R2: TCTAGATTACTTGTACAGCTCGTCCATGCC  F1: AGATCTATGGAGAGCTTTGCGCCGG  R1: GAATTCAGGCACCACTACCAATTGCGTAA  F2: GAATTCATTTCAATGCTCTTCTCGTTTCTG  R2: CTCGAGTTAGGCCCCACCCATGTCG  GATGCCAAAGGCGAGTACGAAGG  GATCGAAATCCGGCCTTGAGCGG |
